# *In silico* DNA barcoding surpasses whole genome sequencing for species identification from vector surveillance pools

**DOI:** 10.1038/s41598-026-39937-y

**Published:** 2026-02-23

**Authors:** C. L. Nascimento, D. P. Tonge, F. Tripet

**Affiliations:** 1 Cambridge University Hospitals - NHS Foundation Trust, Addenbrookes Hospital, Hills Rd, CB2 0QQ Cambridge, UK; 2 School of Life Sciences, Keele University, Newcastle, Staffordshire ST5 5BG UK; 3 School of Medicine, Keele University, Newcastle, Staffordshire ST5 5BG UK; 4 Swiss Tropical and Public Health Institute, Kreuzstrasse 2, 4123 Allschwil, Switzerland; 5 University of Basel, Petersplatz 1, 4001 Basel, Switzerland; 6 School of Life Sciences, Center for Applied Entomology and Parasitology, Keele University, Newcastle, Staffordshire ST5 5BG UK

**Keywords:** Computational biology and bioinformatics, Diseases, Genetics, Microbiology

## Abstract

Mosquito-borne diseases are responsible for over 600,000 deaths annually, mainly in sub-Saharan countries. Mosquito surveillance is a crucial element of vector control programmes to assure they remain effective. This study focused on optimizing the use of the MinION sequencer for interrogating mosquito pools from surveillance programmes, with simplified bioinformatic workflows for use in African laboratories nearer to the field. Mosquito pools were created using different human disease vector species, mimicking possible field trap contents. Some pools were spiked with DNA from the malaria parasite *Plasmodium falciparum* and filarial worm *Brugia malayi*. In the first instance, three pipelines were used to map reads to full reference genomes and their accuracy was compared. Subsequently, mapping reads to full assembled genomes was compared to mapping to a concatenation of diagnostic barcoding sequences for evaluation of relative abundances of mosquito vector species and pathogens. The results show that a combination of Minimap2 with samtools is the most accurate pipeline and a targeted approach preferable to whole genome in estimating species abundance. The MinION device was shown to be effective for interrogating mosquito pools, with moderate training requirements for data analysis. This provides a practical solution to vector surveillance challenges in sub-Saharan Africa.

## Introduction

Mosquito-borne diseases (MBDs) are responsible for high levels of morbidity and mortality, mainly in low-income countries, accounting for 17% of all infectious diseases acquired worldwide^1^. It is estimated that half of the world’s population reside in areas where more than one of these diseases are endemic, and around 80% of the population is at risk of contracting at least one MBDs. According to the World Health Organization (WHO), more than 600,000 deaths occur yearly due to MBDs, and around 96% of these are due to malaria infections^2^. Due to the high incidence and mortality rates, MBDs are among the most researched for disease control, mainly in sub-Saharan Africa (SSA), where the high number of deaths due to malaria coupled with the endemicity of several other MBDs account for a high financial, social and health burden^3,4^. Examples of diseases transmitted by mosquitoes affecting SSA the most are malaria, dengue, yellow fever and lymphatic filariasis. The high mortality, morbidity and economic burden for families and health care systems strengthens the need for more robust vector control and surveillance systems to tackle these diseases^5,6^. The need to strengthen capacity building in SSA countries has been evident by the lack of integration of surveillance tools with decision-making policies^7^. Surveillance is often centralised which leads to inaccurate regional-level assessments and disrupts needed localised actions such as in the event of outbreaks^8^. The Ebola outbreak in the Democratic Republic of Congo (2018-2020) is an example of a successful surveillance strategy that integrated routine genomic testing and public access to the data (stored in government databases)^9^. Taxonomic keys are the gold standard for mosquito identification along with bioassays for detection of insecticide resistance^10,11^. These can be inaccurate/cumbersome, as they rely on human interpretation and often lack standardisation between different assays^12,13^.

Surveillance systems for MBDs should also include routine genomic testing and availability of data for a better integration with vector control measurements in place^14^. Genomics has been proven to be the best tool for a thorough surveillance analysis by being the most accurate technique for species identification and allowing for a fast detection of insecticide resistance^15^. However, its high costs and specialized training required limit its routine use in regional areas. This often results in policy-making decisions that are poorly informed about the specific needs of those areas^16^. Thus, sequencing costs need to be reduced to increase the availability of genomic testing in field-settings and lower-income countries^17^.

Another important factor constraining the integration of genomics tools to MBDs surveillance is the current dependence on expensive equipment such as the NovaSeq Illumina sequencers. These machines or services are still difficult to access in Africa, and users typically queue for weeks to obtain their results. A more promising alternative are the portable miniature sequencers proposed by Oxford Nanopore technologies (ONT)^18^. The ONT MinION sequencer, for example, is a user-friendly and cheaper device (in comparison to other technologies), that can overcome these challenges by allowing sequencing to be done in regional settings and in real-time^19^. Nanopore sequencing has been widely used in genome assembly, analysis of microbial communities from environmental samples as well as entomology studies^20–22^. The barcoding and multiplexing features of the technology allow for the sequencing of multiple samples simultaneously, which reduces costs and analysis turnaround times. To evaluate the value of integrating a device such as MinION in low-income settings in SSA, 5 pools of mosquitoes were created and prepared for whole genome sequencing (WGS) with the purpose of species identification and abundance estimation (e.g. from a single pooled mosquito sample), a major element of mosquito surveillance. Pathogens responsible for high burden MBDs such as malaria and filariasis were added to some of the pools. Five bioinformatics pipelines were used for data analysis: ONT Epi2me Desktop Agent, Centrifuge and 3 variations of Minimap2+Samtools. Minimap2+Samtools were used as default (CLT), filtering reads with quality $>20$ (CLTq20) and filtering only primary reads with quality $>20$ (CLTq20prim). Each pipeline performance was compared in the identification of species and ease of use for low-income settings application. This study also tested the efficiency of using DNA barcoding regions for the detection of both vectors and pathogens from the same mixed samples using an *in silico* preliminary approach. The presented loci sequences and methodology are relevant for the development of a fast and cost-effective laboratory approach to mosquito surveillance. This is ideal for implementation in regional institutions and field locations in lower income regions such as SSA.

## Results

### Data description

Five different mosquito pools were prepared by combining *Aedes aegypti* (*Ae. aegypti*), *Anopheles arabiensis* (*An. arabiensis*), *Anopheles coluzzii* (*An. coluzzii*) and *Culex quinquefasciatus* (*Cx. quinquefasciatus*) mosquito species in different pools (Methods—Table 2) and extracting their DNA. Genomic DNA from *Brugia malayi* (*B. malayi*), *Dirofilaria immitis* (*D. immitis*) and *Plasmodium falciparum* (*P. falciparum*) was added in the last 2 pools. All samples were sequenced using nanopore’s MinION flow cell (Oxford Nanopore Technologies; Ligation sequencing kit SQK-LSK110). Quality control was assessed with Nanoplot software (Table 1). Fastq reads were mapped to the reference genome using CLT, CLTq20, CLTq20prim, Epi2meAgent and classified by Centrifuge (number of mapped reads in Supplementary materials—Tables S1–S5, respectively).

**Table 1:** Quality control of sequencing reads from pools 1-5.

Pool	Number of reads	Mean read length (kbp)	N50 read length (kbp)	Average Phred score	Reads> Q10 (%)	Reads> Q15 (%)
1	8520993	1.38	3.14	13.2	92.80	19.00
2	8403785	1.32	3.01	13.2	92.20	19.10
3	7024751	1.70	3.01	13.2	90.00	15.70
4	11105474	0.58	0.73	12.7	90.10	11.50
5	8297282	0.85	1.12	13.1	91.50	17.60

### Whole genome read mapping approaches and species abundance estimation

**Fig. 1:**
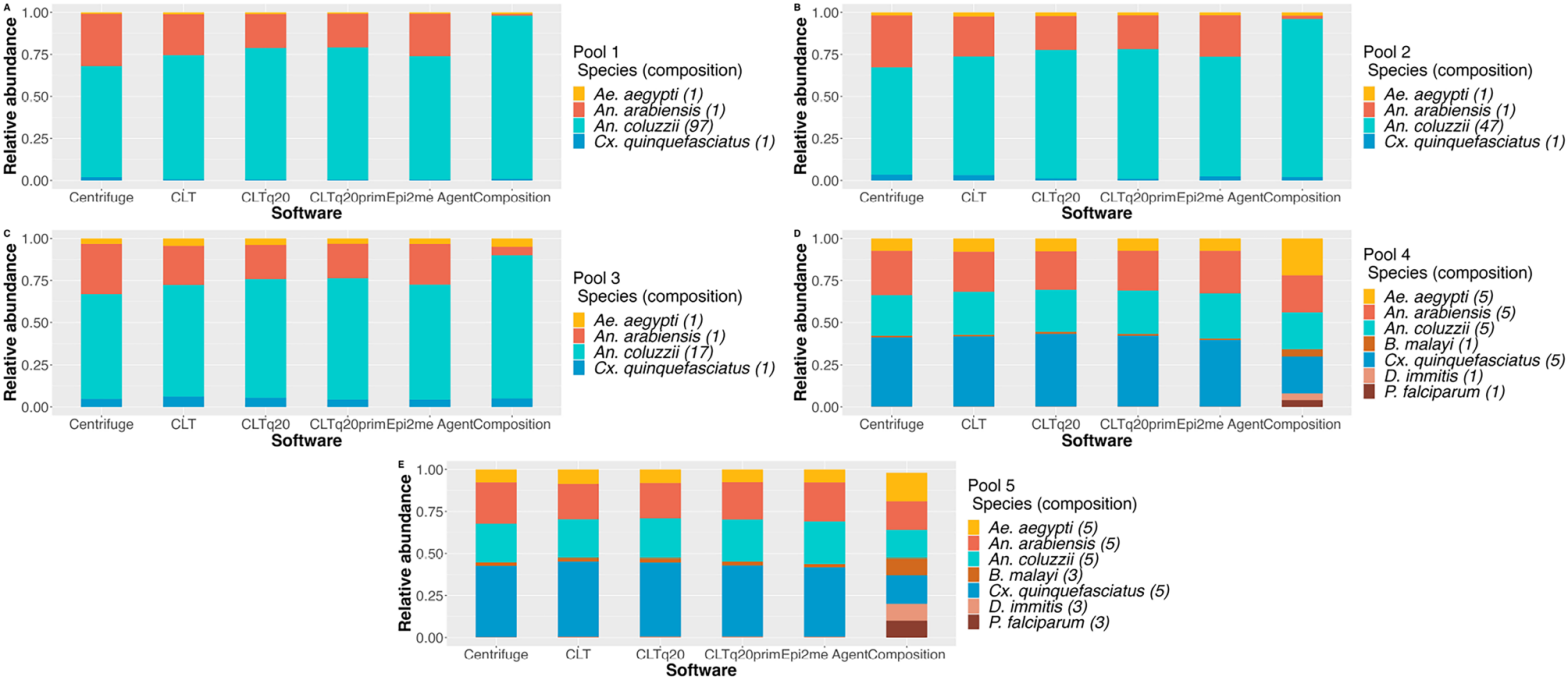
Relative species’ abundance in each pool estimated by each pipeline. Values of RA for each species (represented by different colors) are stacked in each bar corresponding to the estimations done by one pipeline in a total of 5. The values in the stack bar ’Composition’ correspond to the species true ratio in the pools. (**A**–**E**) Species abundance in pools 1–5, respectively. Graphic originated with R software (version 2022.12.0+353) ggplot2, readxl, tidyverse and ggpubr packages (all version 4.2.0).

In pools 1–3, in which *An. coluzzii* was the most abundant species, all pipelines attributed the highest relative abundance (RA) to this species (values between 0.60 and 0.78—Fig. 1). These values were lower than the actual pool composition (0.97, 0.96 and 0.85 for pools 1–3, respectively). The values of RA for *Aedes* and *Culex* species were similar to the pool’s ratio (0.01, 0.02 and 0.08 for pools 1–3, respectively). *An. arabiensis* was over-estimated in all pools by all pipelines used. The RA values varied between 0.20 and 0.31 in the 3 pools, while its actual pool representation was 0.01, 0.02 and 0.05 (pools 1–3). In pools 4 and 5, calculated values of RA for *An. arabiensis* were similar for *An. coluzzii* (varying between 0.21 and 0.26), near the pools’ actual composition (0.22 and 0.17 for pools 4 and 5, respectively). *Ae. aegypti* was under-represented at about 1/3 of its true expected ratio (values obtained of 0.07–0.09) and, inversely, *Cx. quinquefasciatus* was over-estimated twice as much as its ratio in the pools (abundance of 0.40 to 0.45 when comparing to 0.22 and 0.17 ratio in pools 4 and 5, respectively). From all parasite gDNA used in pool 4, only *B. malayi* was detected by the 5 different pipelines although under-represented (abundance of 0.01 comparing to the actual value of 0.04). In pool 5, all pipelines identified *B. malayi* and *D. immitis*. The values were lower (0.02 and 0.01, respectively) than their proportion in the pool (0.10). The RA values for *P. falciparum* in pools 4 and 5 did not reach 0.001 (it was determined as unidentified). 

**Fig. 2:**
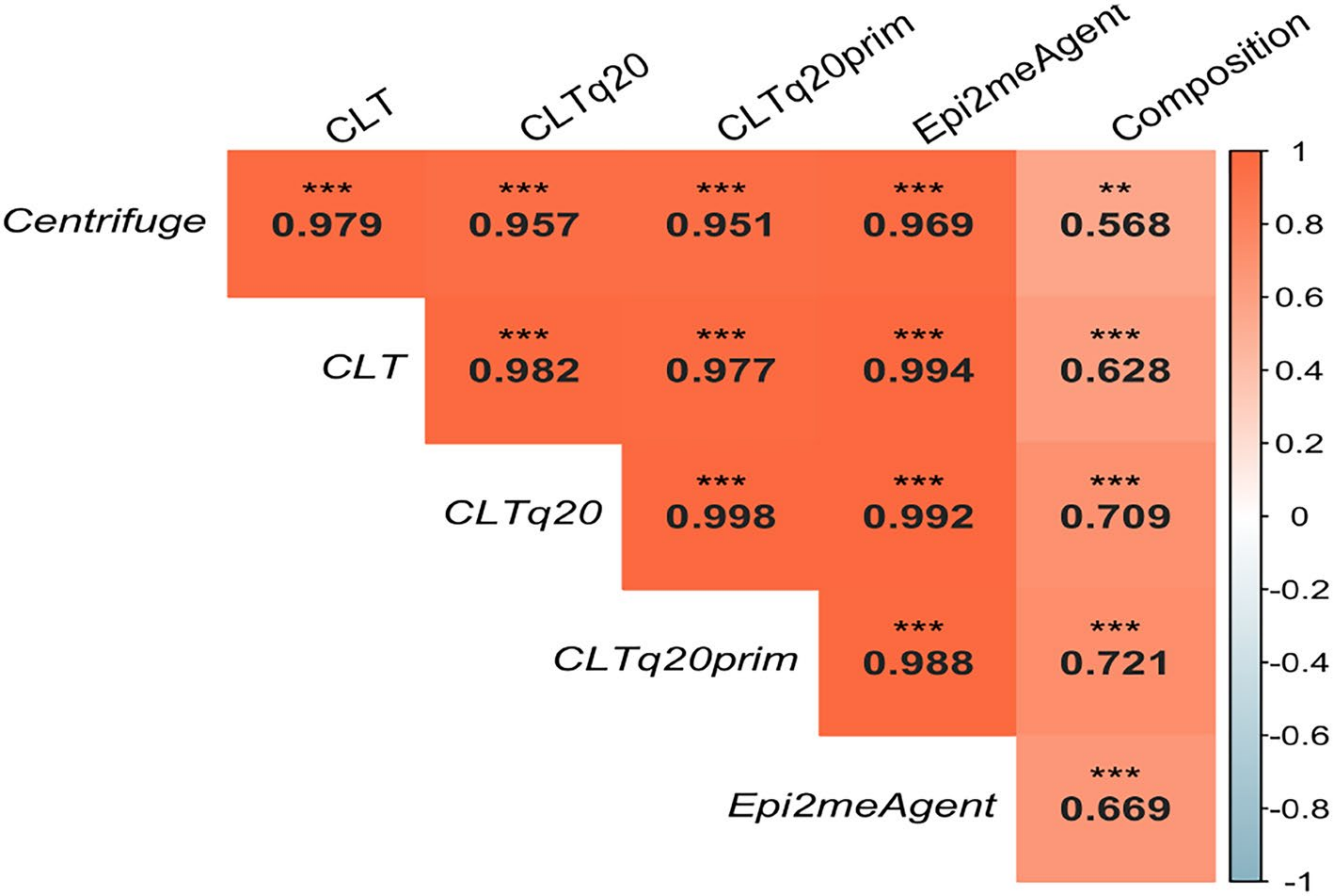
Spearman’s rank correlation values between RA calculated by each software and the pools’ true composition. Correlation values are represented for each association with the strongest positive correlations (near 1) in orange, stronger negative correlation (near – 1) in blue and no correlation (near 0) in white. Significance is represented by 2 or 3 asterisks which represent P-value < 0.01 and < 0.001, respectively. The statistical analysis (and plot generated) were performed using *R* software (version 2022.12.0+353) - readxl, car, stats, psych, and FSA packages (all version 4.2.0).

From the different pipelines used, CLTq20prim was the best performer at estimating species abundance in the different pools (Spearman’s rank correlation of 0.721 - P-value <0.05—Fig. 2). All correlations measured were statistically significant. The difference between correlations was not statistically significant (P-value >0.05).

#### Sequencing coverage and depth

The higher values of sequencing depth were obtained for the mitochondrial genome (ranged from approximately 20× to 1000x for all species except *Aedes*—Supplementary materials—Table S6). For the remaining chromosomes, coverage was between 40 and 90% for both *Anopheles* spp. while *Culex* spp. had values between 10 and 20% in the first two pools and only surpassing 50% in pools 3-5, reaching a maximum of 78%. *Aedes* spp. had the lowest coverage, with a maximum of 40% in pools 4 and 5 and around 20, 30 and 50% in samples 1-3. Pools 4 and 5 yielded a maximum sequencing depth of 0.63x, 1.90x, 2.68x and 3.24x for *Ae. aegypti*, *An. arabiensis*, *An. coluzzii* and *Cx. quinquefasciatus*, respectively. These values corresponded to the depth achieved when sequencing 5 mosquitoes of each species. Sequencing depth for all parasites ranged between 0.00x and 0.43x in both pools, with *D. immitis* achieving the highest. The genome coverage for all mosquitoes was also higher within the mitochondrial chromosome in comparison to the others with percentages of coverage of approximately 100 except for *Ae. aegypti* - maximum of 63.24 and 0.00 in pool 5. *B. malayi* had coverages of 16 and 24% in pools 4 and 5, while *D. immitis* was 3 times lower (5 and 9%). *P. falciparum* aligned reads did not reach 1% coverage (also in congruence with the previous consideration of being unidentified).

### Species’ DNA quantity effect in RA estimation

To verify whether different body sizes between mosquitoes could have an impact on the previous results, 12 mosquito extractions were performed for each mosquito species. The concentrations obtained for each mosquito species and the standard deviation were: *Ae. aegypti* 38.53 ng/$\mu$L (± 13.23), *An. arabiensis* 20.94 ng/$\mu$L (± 9.80), *An. coluzzii* 23.38 ng/$\mu$L (± 7.01), and *Cx. quinquefasciatus* 52.53 ng/$\mu$L (± 20.47). Only between *Ae. aegypti*–*Cx. quinquefasciatus* and *An. arabiensis*–*An. coluzzii* was the mean DNA concentration not statistically different (*P* value > 0.05).

**Fig. 3:**
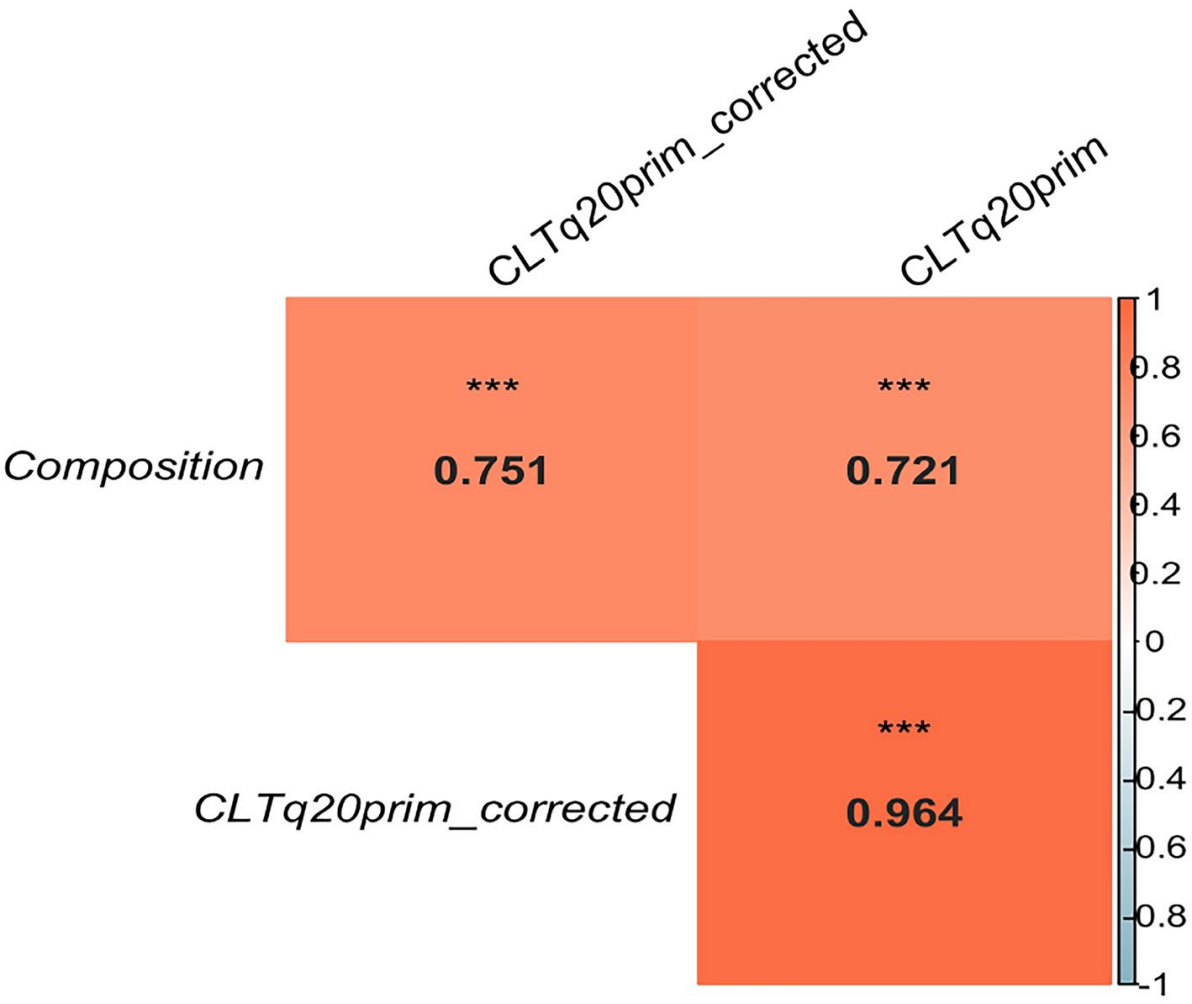
Spearman’s rank correlation between RA before and after correction for species’ DNA concentration after extraction. Correlation values are represented for each association. The stronger positive correlations (near 1) in orange, stronger negative correlations (near − 1) in blue and no correlation (near 0) in white. Significance is represented by 3 asterisks which represent P-value < 0.001. The statistical analysis (and plot generated) were performed using *R* software (version 2022.12.0+353)—readxl, car, stats, psych, and FSA packages (all version 4.2.0).

The values of RA (calculated with CLTq20prim) were corrected for DNA concentration and the correlation with the actual values of the pools was calculated. The Spearman correlation value increased from 0.72 to 0.75 (Fig. 3). The difference was not statistically significant (P-value > 0.05).

### Whole genome mapping and distinguishing members of the *An. gambiae* complex

**Fig. 4:**
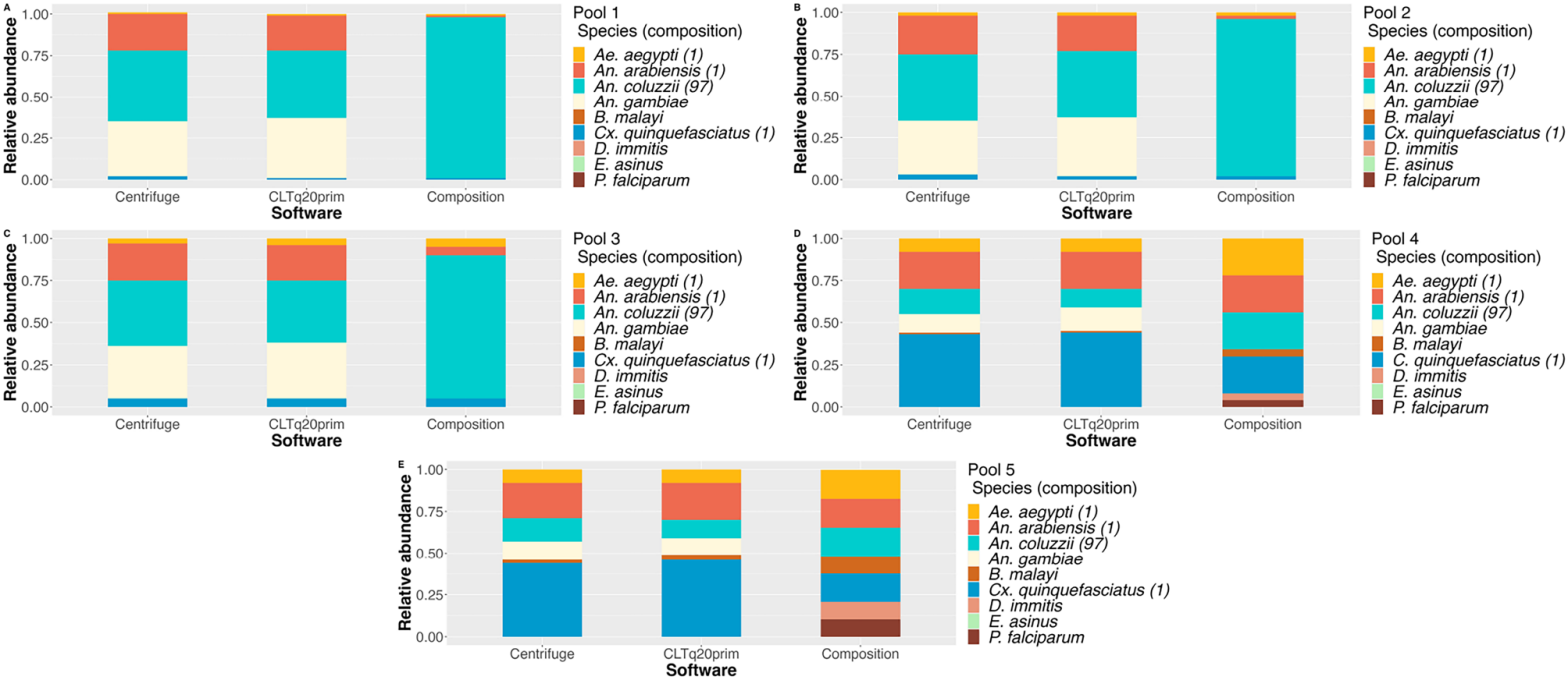
Relative species’ abundance in each pool estimated by each pipeline when mapping reads to a control reference genome. Values of RA for each species (represented by different colors) are stacked in each bar corresponding to the estimations done by one pipeline in a total of 5. The values in the stack bar ’Composition’ correspond to the species true ratio in the pools. (**A**–**E**) Species abundance in pools 1–5, respectively. Graphic originated with R software (version 2022.12.0+353) ggplot2, readxl, tidyverse and ggpubr packages (all version 4.2.0).

All reads from the 5 different sequencing runs were mapped to a single control reference genome to evaluate whether the reads would align to species not present in the pools (Supplementary materials—Tables S7, S8). The estimated values were similar to the ones obtained when using references composed only of species present in the pool for *Ae. aegypti*, *An. arabiensis* and *Cx. quinquefasciatus* (Fig. 4). *An. coluzzii* was represented with the highest ratio of all species in pools 1–3 but with lower RA values. The reads mapped to *An. gambiae* reach a higher RA than *An. arabiensis* and at approximately 1/3 of *An. coluzzii*. Centrifuge classified fewer reads of *An. gambiae* origin, comparing to CLTq20prim, although with similar numbers. All pipelines assigned reads to *Equus asinus* (*E. asinus*); however, its RA was not significant (< 0.001). No statistical tests were performed, as both Software assigned reads to species not present in the pools.

#### Sequencing coverage and depth

When comparing the sequencing depth and coverage for the species not present in the pool with the ones present, it was shown that *An. gambiae* had similar results to *An. coluzzii* in all pools and higher sequencing depth than *An. arabiensis* (Supplementary materials—Table S9). Regarding the parasites and *E. asinus*, the depth was approximately zero in all pools (when parasites not present) and the coverage below 0.4%. Maximum sequencing depth for all vector species in pools 4–5 was 1.63x and coverage ranged between 35 and 80%. Mitochondrial chromosome coverage was close to 100% for all mosquitoes in all pools except for *Ae. aegypti* with an average of 20%, reaching 60% in pool 3 (Supplemenmtary materials - Table S9).

### Mapping of whole genome sequencing reads from mixed pools to key target regions

**Fig. 5:**
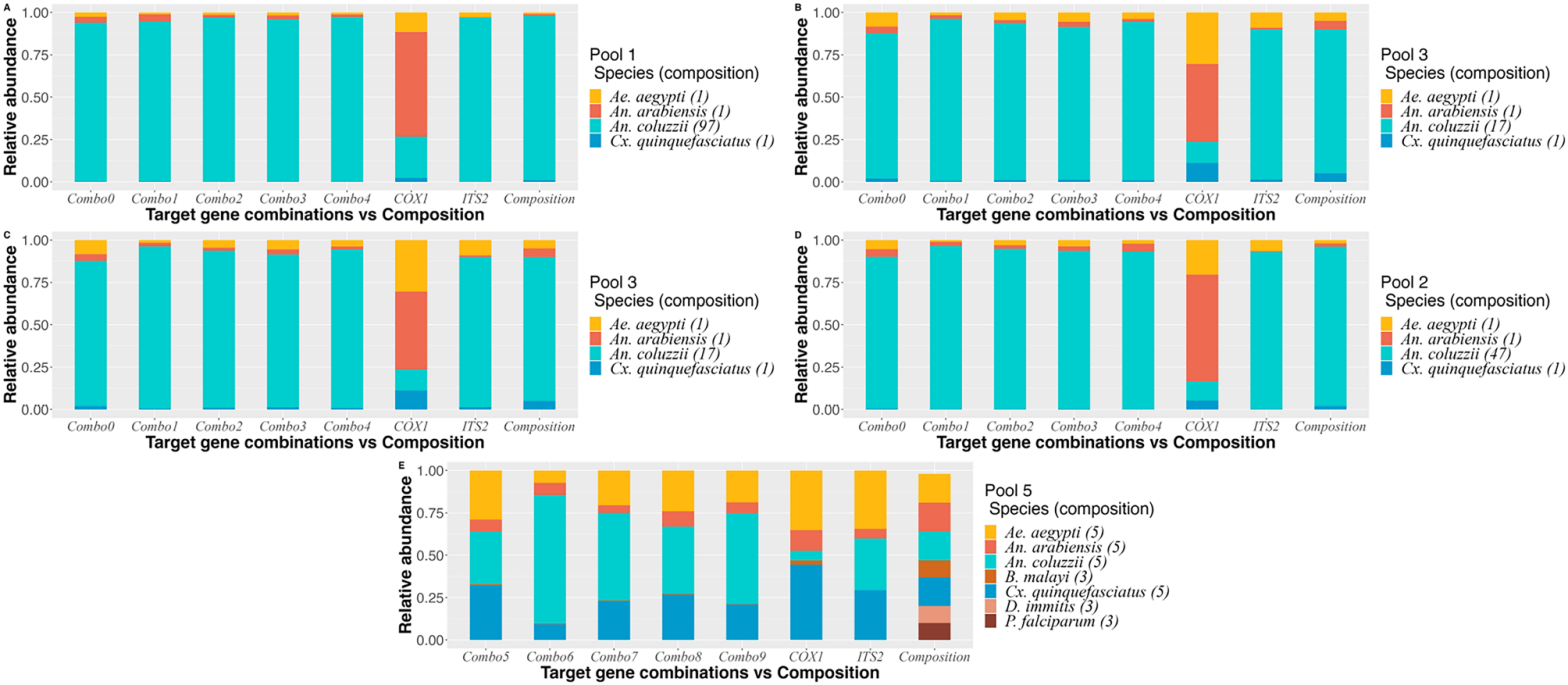
Relative species abundance when mapping WGS reads to different combinations of target regions. Graphical representation of species RA in pools 1–5. Each graph contains a stack bar titled composition which corresponds to each species ratio in the prepared sample. (**A**–**C**) Species abundance in pools 1–3, respectively, analysed with combo 0, 1, 2, 3, 4, COX1, COX1(ac)_IGS, COX1(ac)_SINE and ITS2 target loci. D-E) Species abundance in pools 4-5, respectively, analysed with combo 5, 6, 7, 8, COX1, COX1(ac)_IGS, COX1(ac)_SINE and ITS2 target loci. Graphic originated with R software (version 2022.12.0+353) ggplot2, readxl, tidyverse and ggpubr packages (all version 4.2.0).

The sequencing from the 5 different pools were also mapped to combinations of target regions (Table 3) for vector and pathogen identification (Supplementary materials - Table S10). The calculated RA of *An. coluzzii* was similar to its proportion in pool 1 (Fig. 5). In the same pool, *Cx. quinquefasciatus* was under-represented never reaching 0.01 in any combination used. *An. arabiensis* values obtained ranged from 0.01 to 0.04 (real ratio 0.01) similarly to what obtained for *Ae. aegypti* (0.01–0.03). *Ae. aegypti* was estimated at a similar ratio in pools 2 and 3 (between 0.01–0.05 and 0.02–0.08, respectively) varying slightly under or higher than the pools’ true composition values (0.02 and 0.05, respectively). *An. arabiensis* abundance estimation for pool 2 varied between 0.02 and 0.04, which was the same or slightly higher than the actual prepared ratio, and was under-represented in pool 3 (under 0.05). *An. coluzzii* estimated values, for all combos, ranged from 0.90–0.96 and 0.86–0.96 for pools 2 and 3 which was within the correct range for pool 2 (0.94) and slightly over-estimated in pool 3 (comparing to the value of 0.85 in the pool’s composition). *Cx. quinquefasciatus* was under-represented in both pools 2 and 3, with values ranging between 0.00–0.01 and 0.01–0.02, respectively, when comparing to its ration in the samples (0.02 and 0.05). In pools 4-5, the calculated values of RA for *Ae. aegypti* were lower than the pools’ actual composition when using combo 6. The pipeline estimated its abundance as 0.10 and 0.07, while its ration in the pools was 0.22 and 0.17, respectively. The other combos estimated its abundance between 0.21 and 0.28 which was similar to its abundance of 0.22 in pool 4. For pool 5, the combos estimated this vector abundance being between 0.18 and 0.29, which was higher than what prepared. *An. arabiensis* was estimated between 0.05 and 0.09 of the total abundance for pools 4 and 5 which was an under-representation of the species ratio in both pools’ composition (0.22 and 0.17 respectively). *An. coluzzii* was always over-represented in pools 4 and 5, with combo 5 and 9 giving the most approximate RA value of 0.33/0.34 for pool 4 and 0.30/0.39 for pool 5, when comparing to 0.22 and 0.17 (its ration in the pools). All other combos calculated its abundance between 0.40–0.72 and 0.51–0.76 for both pools, respectively. *Cx. quinquefasciatus* was under-represented in both pools only when using combo 6 (calculated value of 0.10 for both pools in comparison to the target 0.22 and 0.17). When using the other combos, the calculated abundances ranged between 0.21 and 0.34. Only *B. malayi* was detected at 0.01 when using combo 8 in pool 5 and at lower quantity with the remaining combos. *D. immitis* and *P. falciparum* were always estimated with an abundance under 0.01 in both pools and for this reason were classified as unidentified.

**Fig. 6:**
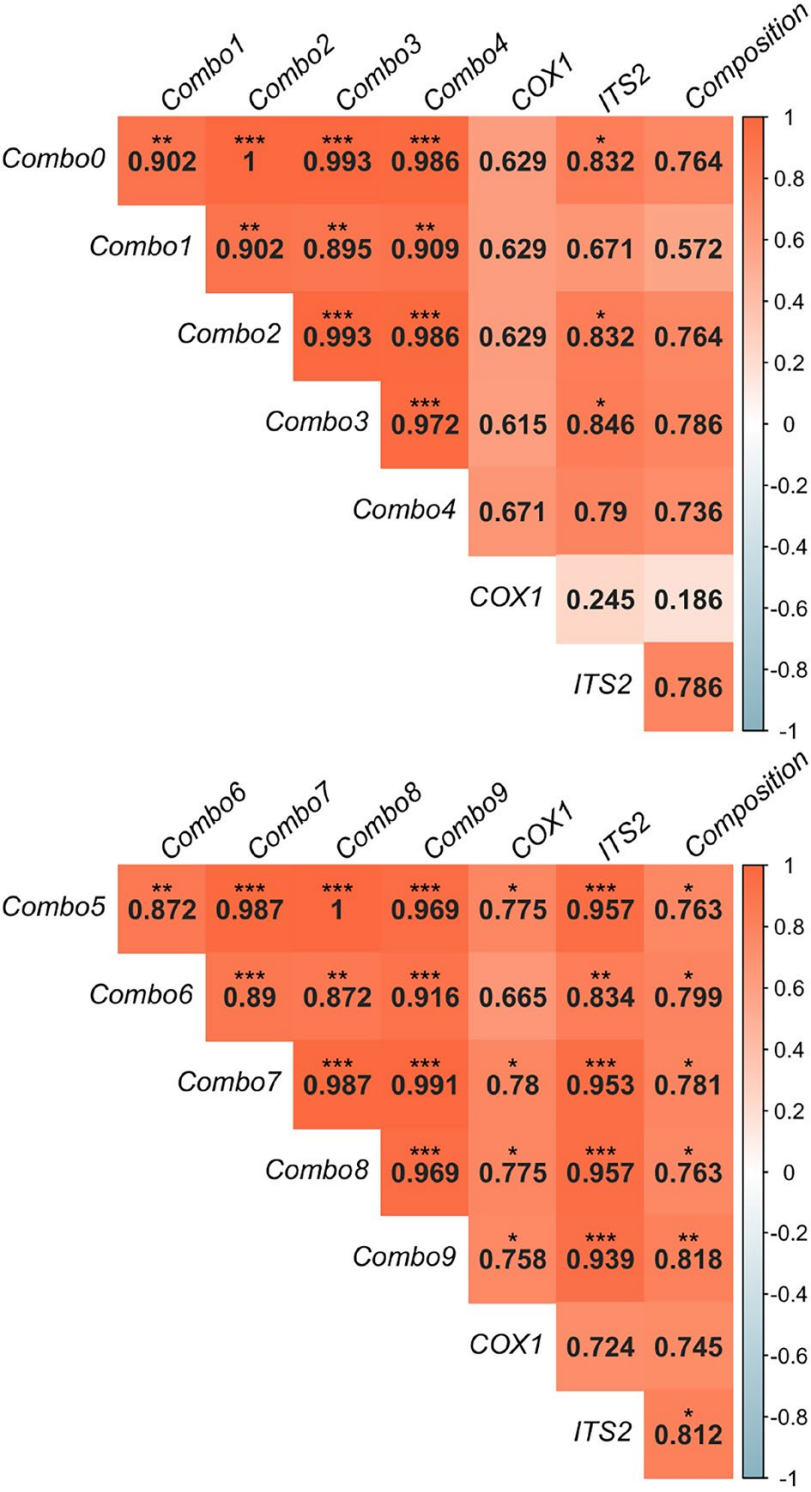
Spearman’s rank correlation between RAs obtained when using different loci combinations and the pools’ true composition. Correlation values are represented for each association with the strongest positive correlations (near 1) in orange, stronger negative correlation (near − 1) in blue and no correlation (near 0) in white. Significance is represented by 1, 2 or 3 asterisks which represent P-value < 0.05, 0.01 and 0.001 respectively. The statistical analysis (and plot generated) were performed using *R* software (version 2022.12.0+353)—readxl, car, stats, psych, and FSA packages (all version 4.2.0).

The correlation between calculated RA values per Software and the pools true values was measured. The values of RA obtained when using COX1 and ITS2, individually, were also included in the calculation. As different regions were included in the combinations used for pools 1–3 and 4–5 (depending on the presence of pathogens), both sets of pools were analysed individually. For pools 1-3, values of species abundance correlate the most with the pool’s true composition, when mapping reads to combo 3 and ITS2 (Spearman’s rank correlation value of 0.79—Fig. 6). The second best performers were combos 0 and 2 (0.76) and finally combo 4 (0.74). When mapping reads to combo 1, the correlation value did not reach 0.60 and almost no correlation was observed when using the COX1 locus individually (0.19). None of the correlations was statistically significant (P-value > 0.05). In pools 4 and 5, the highest correlation with the actual species proportions was obtained when mapping reads to combo 8 (0.82) followed by when using the ITS2 region individually (0.81). Combo 6 followed with a correlation value of 0.8. All correlations were statistically significant (P-value < 0.05) except for values obtained when using the COX1 region alone.

#### Sequencing coverage and depth

When considering target identification loci, sequencing coverage reached 100% for almost all species (Supplementary materials—Table S11). The only exception being the reads mapped to the SINEs200 regions of both *Anopheles* species in pool 4. The highest values of sequencing depth (> 10000x) were obtained when mapping the reads to the ITS2, SINEs200 and IGS regions of *An. coluzzii* in all pools. Depth values for the ITS2 locus from *Ae. aegypti* ranged from 390 to 1444x in all pools, and were the second highest range (with *Cx. quinquefasciatus* ITS2 locus showing values slightly above in pools 4–5). The lowest values were obtained for the filariae and *An. arabiensis* SINEs200 in all pools (maximum of 15x). In pools 1–3, reads mapped to the COX1 region had a higher depth for *An. arabiensis* (between 570 and 750x), whereas for pools 4-5, *Ae. aegypti* led with 220–250x.

Sequencing coverage values ranged between 70 and 100% for all loci and species in all pools.

### Targeted mapping and false positives

**Fig. 7:**
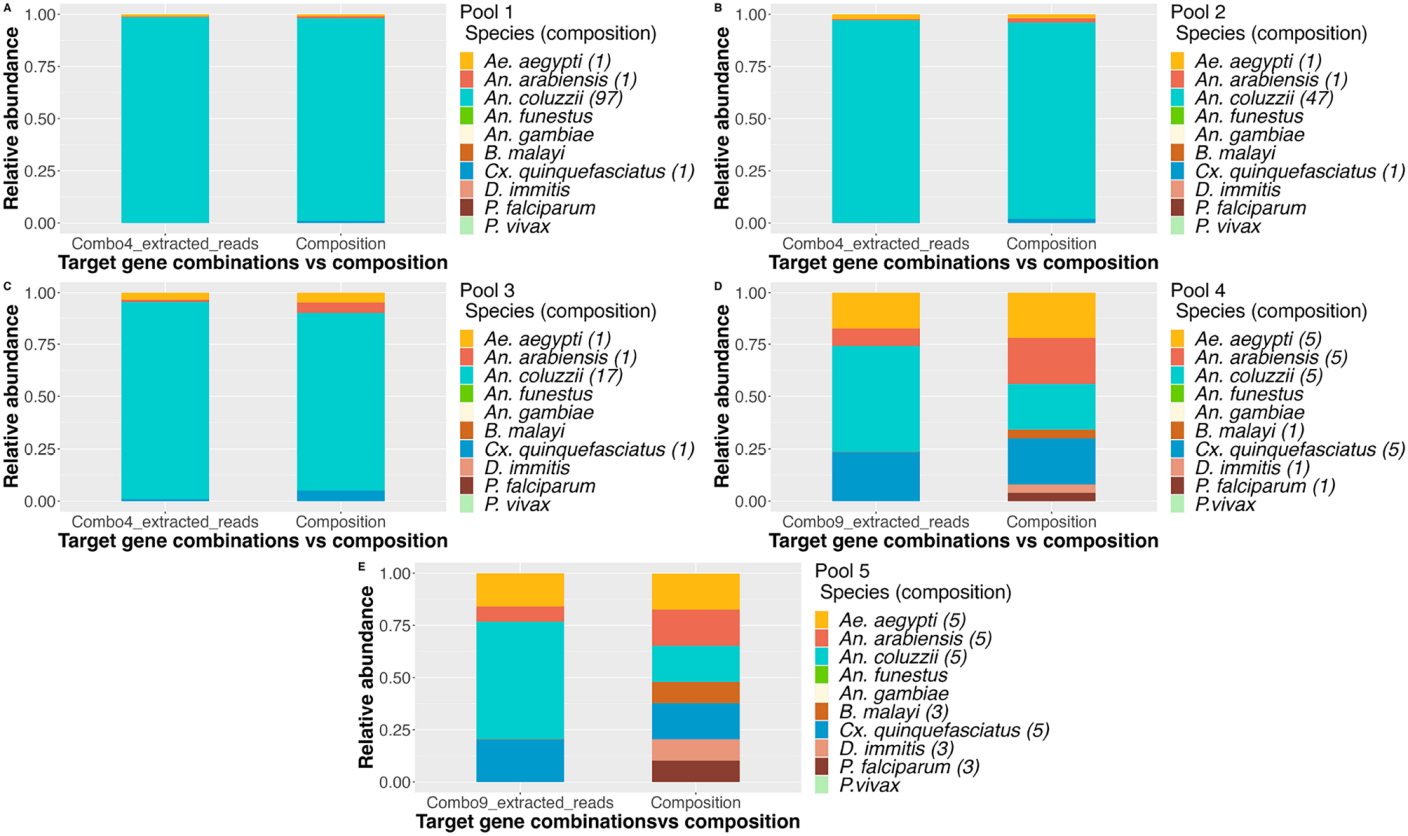
RA of species when mapping target extracted reads to control reference loci. WGS mapped reads to combo 4 (pools 1–3) and combo 9 (pools 4–5) were extracted and mapped to a control reference (composed of all target loci for species identification) and RA determined. Each graph contains a stack bar titled composition which corresponds to each species ratio in the prepared sample. (**A**–**C**) Species abundance in pools 1–3. (**D**, **E**) Species abundance in pools 4–5. Graphic originated with R software (version 2022.12.0+353) ggplot2, readxl, tidyverse and ggpubr packages (all version 4.2.0).

Whole genome sequencing reads were also mapped to key regions of species not present in the pool (Supplementary materials—Table S12). The same species were used as mentioned before for fully assembled genomes. In the first 3 pools, *An. coluzzii* was correctly estimated as the most abundant species (Fig. 7). *Cx. quinquefasciatus* was under-estimated as well as *An. arabiensis*. *Ae. aegypti* was represented in an equivalent ratio as expected. *Cx. quinquefasciatus* was detected at very low levels. No reads were mapped to *An. funestus*, *P. falciparum* or *P. vivax*. In pools 4 and 5, all vectors were under-estimated with the exception of *An. coluzzii*, which was represented as the most abundant species. Calculated RA values for *Cx. quinquefasciatus* are equivalent to the pools’ true composition and slightly lower for *Ae. aegypti*. *An. arabiensis* was determined in approximately 1/3 of what prepared in the samples. Although some reads were mapped to *An. gambiae*, estimated values of RA were lower than 0.0001, which in the overall abundance of the pools was deemed non-significant.

#### Sequencing coverage and depth

The highest values of sequencing depth were obtained for reads mapped to *An. coluzzii* ITS2, SINEs200 and IGS target regions, for pools 1–3 (18000x–25000x—Supplementary materials—Table S13). In these pools, values below 100x were obtained for *An. arabiensis* SINEs200, and *Cx. quinquefasciatus* ITS2 (the latter not observed for pool3). Although *An. gambiae* RA abundance values were too low to be deemed significant, they were originated from reads mapped to its COX1 reference in all pools (except pool 3), ITS2 in pool 2 and IGS in pool 3. The values of sequencing depth never reached 7x. Sequencing coverage in the 3 pools was 100% for reads mapped to all loci with the exception of the reads mapped to *An. gambiae* (between 74-96%). In pools 4 and 5, values of sequencing above 1000x were obtained for *An. coluzzii* ITS2, SINEs200 and IGS; for *Ae. aegypti* ITS2 (pool 4); and *Cx. quinquefasciatus* ITS2. Values below 100x for reads mapped to vector references were obtained for *An. arabiensis* COX1 and IGS; for *An. gambiae* and for *An. coluzzii* COX1 regions. Both filarias achieved sequencing depth values of 40x and 4x for *B. malayi* and *D. immitis*, respectively. Sequencing coverage was below 100% for the IGS regions of *An. coluzzii and arabiensis* (pool 4); for the COX1 regions of *An. gambiae*, *D. immitis* and *An. coluzzii* (pool 5). The coverage was 100% for all other regions and pools.

### Price estimation

Five different pools were sequenced individually in 5 MinION flow cells. The overall price of the sequencing experiments (outsourced) was £4500 (GBP). The costs of DNA extraction were not reported, as this varies according to the DNA extraction method implemented in each laboratory, being easily adjusted. The price estimation for the competitor Illumina would be approximately two times more (including library preparation and sequencing reagents). An important note is that only the NovaSeq equipment allows WGS analysis. The supplier’s website does not describe which reagent is the best for WGS but the prices are higher than for the more affordable sequencers such MiniSeq (accessed in https://emea.illumina.com /systems /sequencing-platforms /miniseq /order-miniseq.html). The MiniSeq Illumina sequencer (one of the most affordable available) costs around $\pounds$37K (GBP) (only allowing for short read sequencing) and the ONT MinION Mk1D Pack is approximately $\pounds$3.7K (GBP) (including flow cells, reagents and a sequencing kit) and is suitable for WGS. Software for Illumina sequencing needs to be purchased, which has a cost of $\pounds$400 (GBP) annually while Nanopore MinKNOW software is free. The ONT MinION sequencer offers a cheaper solution for the same methodology.

## Discussion

This study aimed to evaluate the use of ONT whole-genome sequencing for pooled mosquito surveillance samples. In addition, it assessed the use of *in silico* target loci as molecular biomarkers in preparation for a potential amplicon-based species identification strategy. The use of target loci was shown to be more effective in the estimation of species abundance when compared with mapping reads to fully assembled reference genomes. This approach not only was more accurate in differentiating between closely related species (such as the members of *An. gambiae* complex) but also did not identify species not present in the pools (false positives), an issue with the WG mapping approach. This showed that, although a WGS approach may be technically more straightforward, targeted approaches are preferable moving forward. This *in silico* proof of concept confirmed that a PCR amplicon-based approach would be more accurate for interrogating mixed mosquito pools from, for example, field trap contents. The use of sequencing techniques is associated with high quality requirements of DNA purity. When considering the settings from regional laboratories in SSA, ethanol preservation is often the standard used for mosquito storage. The samples prepared in this study were preserved with ethanol for a better accuracy and representation. In the field, samples collected from traps are exposed to high temperature and humidity conditions which possibly lead to DNA degradation. The samples used for WGS, here described, have a lower value of DNA purity than required. This was ideal to investigate what is likely expected when preforming WGS in field settings. The sequencing technology was still able to identify vectors and parasites in mixed pools, particularly when in low numbers, showing good limits of detection. These *in silico* results will need to be validated using actual PCR-generated amplicon pools of selected sequences. The amplicons can be multiplexed using barcoding of libraries to improve cost effectiveness. Target regions such as COX1 and ITS2 have been widely use in metagenomics and mosquito identification for their conserved regions flanking individual sequences species’ specific^23–25^. By using WGS reads and mapping them to these specific regions, we observed that the ITS2 locus was more efficient in differentiating the 4 different species of mosquitoes than COX1. Similarly to what was observed in previous studies, the COX1 region did not allow for the distinction of members of the *An. gambiae* complex^21^. The same study reported that the COX1 locus differentiated between *Aedes*, *Culex* and *Anopheles* vectors, however, we observed the contrary, particularly when vector species are present in the some ratio in mixed pools. For this reason, we determined that this target region was not accurate in estimating the mosquito abundances in mixed pools (although a PCR-based strategy is needed for confirmation). Although more efficient in differentiating species, the ITS2 reference loci used were not successful in determining the exact abundances between *An. arabiensis* and *An. coluzzii*. This was also not congruent with what obtained elsewhere^26^.

The sequences used might be one of the reasons for the inefficiency in differentiating members of the *An. gambiae* complex. The sequencing technologies used to assembly most of the references used also have gaps in efficiency, due to the limited number of base pairs they can sequencing per read as well as to the low efficiency in assigning nucleotides in homopolymeric regions^27,28^. For implementation of this technology in SSA, it is crucial that only species present in the pools are identified. Thus the *in silico* evaluation of a targeted approach strongly suggest that is the method of choice for identifying vectors and pathogens from mixed pools. For field deployment, this would imply that mosquito communities are well described such that target sequences can be designed for medically-relevant mosquito species. The findings to support this were a higher correlation between RAs estimated and the pools’ different composition and the identification of species in pools, only when present. A targeted approach accurately led to a decrease in the abundance estimation of *An. arabiensis* (in pools 1–3). However, the values remained low when this vector was present in the same quantities as the other 3. One of the downfalls of the *in silico* targeted approach was that it relied on the low sequencing depths achieved when sequencing genomic DNA, which might have impacted its efficiency. The ITS2 region and combo 3 (combination of ITS2, COX1, IGS) were the best in discriminating species and estimating their abundance from mixed pools. A combination of all target regions (combo 9) is a possible option with good results as well. Further studies are needed to confirm the power of this targeted approach, and to evaluate the possible bias originated by amplicon-base approaches. As the PCR amplification generates a large number of copies of the same target, low starting quantities of DNA could be used. By using target sequencings, mapping will be less computing intensive, making it possible to analyse results locally, without the need to use the Nanopore online platform, which is more relevant to field deployability. Overall, the *in silico* results suggested that an amplicon-based approach should be cost effective and would allow the effective detection of mosquito species and pathogens even if present at low to very low prevalence.

## Methods

### Pool design for whole genome sequencing

**Table 2:** Description of the number of vectors and pathogens in pools for WGS analysis.

Pool	Species	Number	Total	Composition
1	*Ae. aegypti*	1	100	0.01
	*An. arabiensis*	1		0.01
	*An. coluzzii*	97		0.97
	*Cx. quinquefasciatus*	1		0.01
2	*Ae. aegypti*	1	50	0.02
	*An. arabiensis*	1		0.02
	*An. coluzzii*	47		0.94
	*Cx. quinquefasciatus*	1		0.02
3	*Ae. aegypti*	1	20	0.05
	*An. arabiensis*	1		0.05
	*An. coluzzii*	17		0.85
	*Cx. quinquefasciatus*	1		0.05
4	*Ae. aegypti*	5	23	0.22
	*An. arabiensis*	5		0.22
	*An. coluzzii*	5		0.22
	*Cx. quinquefasciatus*	5		0.22
	*B. malayi*	1		0.04
	*D. immitis*	1		0.04
	*P. falciparum*	1		0.04
5	*Ae. aegypti*	5	29	0.17
	*An. arabiensis*	5		0.17
	*An. coluzzii*	5		0.17
	*Cx. quinquefasciatus*	5		0.17
	*B. malayi*	3		0.10
	*D. immitis*	3		0.10
	*P. falciparum*	3		0.10

**Table 3:** Combinations of target identification loci for read mapping.

Target regions (species)	Combination
Pools 1-3	
COX1 + ITS2 (all vectors)	Combo 0
COX1 + SINEs200 + IGS	Combo 1
COX1 + ITS2 + SINEs200	Combo 2
COX1 + ITS2 + IGS	Combo 3
COX1 + ITS2 + SINEs200 + IGS	Combo 4
Pools 4-5	
COX1 + ITS2 + 18srRNA	Combo 5
COX1 + SINEs200 + IGS + 18srRNA	Combo 6
COX1 + ITS2 + SINEs200 + 18srRNA	Combo 7
COX1 + ITS2 + IGS + 18srRNA	Combo 8
COX1 + ITS2 + SINEs200 + IGS + 18srRNA	Combo 9

#### Species collection

Four different mosquito strains were reared in the insectaries of Keele University (Center for Applied Entomology and Parasitology) by technicians and included: *Ae. aegypti* strain New Orleans, *An. arabiensis* strain Rufisque, *An. coluzzii* strain VK and *Cx. quinquefasciatus* strain JHB. The mosquitoes were kept at 27±2°C, 70±5% relative humidity and in a 12 hour light/dark cycle. Trays with water were used for the larval stages (kept at a density of 200/l and fed with ground fish food—Tetra min, Tetra, Melle, Germany). The larva that pupated were transferred to white polypropylene 5L bucket rearing cages (20 cm diameter x 20.5 cm height). Adult mosquito population per cage was maintained at around 600–800 individuals with a 5% glucose solution provided at all times. The adult mosquitoes were aspirated from the cages and left at -20°C for 20 min and subsequently transferred to a 50 ml centrifuge tube containing a 75% ethanol solution, which was kept at – 20°C^29^. Genomic DNA from the three parasites was obtained from the Biodefense and Emerging Infections Research Resources Repository (beiresources): *B. malayi* strain FR3 (item number: NR-42491, batch number: 62359777), *D. immitis* strain Missouri 2005 (item number: NR-44348, batch number: 63425218) and *P. falciparum* strain Dd2 (item number: MRA-156G, batch number: 70034409). All samples were treated with RNase A and diluted in TE buffer (1 mM Tris-HCl, 0.1 mM EDTA, pH: 8), provided at a concentration of 50 ng/$\mu$L.

### Genomic DNA extraction

QIAgen DNeasy blood & tissue kit specific for insects with modifications was used for DNA extraction from mosquitoes. The mosquitoes were removed from the ethanol solution and were washed in 1xPBS solution. They were transferred into a 1.5 $\mu$L tube containing 180 $\mu$L of PBS. The DNA extraction steps were followed according to the manufacturer’s standard instructions. The resulting DNA eluate was stored at -20 °C. Pool extractions were analysed using Nanodrop 2000 for concentration and purity determination. Each sample was analysed 3 times and the mean value calculated. The DNA extractions were also added into a 0.7% agarose gel for visualization of DNA fragment length (Supplementary materials - Figure S1). One hundred ng of each sample was added to the gel. The samples and ladder (NEB Quick-loadpurple Extended 1Kb plus) were subjected to electrical current for 2.5 h at 90 V 400 mA.

#### Sample preparation

Pool 1 was prepared by adding together five individual extractions of 20 mosquitoes each. Four extractions were composed of only *An. coluzzii* vectors. The fifth extraction contained 17 *An. coluzzii*, 1 *Ae. aegypti*, 1 *An. arabiensis* and 1 *Cx. quinquefasciatus* mosquitoes. Pool 2 combined two extractions of 20 *An. coluzzii* and another with 7 *An. coluzzii*, 1 *Ae. aegypti*, 1 *An. arabiensis* and 1 *Cx. quinquefasciatus* mosquitoes. Pool 3 was prepared as a single extraction of 17 *An. coluzzii*, 1 *An. arabiensis* and 1 *Cx. quinquefasciatus* mosquitoes. Pools 4 and 5 were prepared with a single extraction of 20 mosquitoes (5 mosquitoes of each species mentioned previously). No sex identification was performed when preparing the pools, these consist of both male and female mosquitoes.

#### Addition of pathogen genomic DNA

Genomic DNA from *B. malayi*, *D. immitis* and *P. falciparum* was added to pools 4 and 5 (Table 4). The quantity of DNA added to pools 4 and 5 was equivalent to 1 and 3 genome copies of each parasite, respectively. Each genome copy was determined as equivalent to the genome size of that organism, in base pairs. Pools 4 and 5 were quantified in base pair numbers, assuming each individual vector and pathogen in a pool corresponded to 1x its genome size. The percentage of each vector and pathogen in the pool was calculated relative to the total number of base pairs in the pool. The total amount prepared for each pool was 2500 ng. The genomic DNA was provided by the manufacturer in vials of 50 ng/$\mu$L and diluted to a final concentration of 12.5 ng/$\mu$L.

**Table 4:** Quantity of genomic DNA from parasites added to pools 4 and 5.

Species	Genome size (bp)	Total bp (%)	Quantity (ng)	Conc. (ng/$\upmu$L)	Volume ($\upmu$L)
Pool 4					
*B. malayi* (1)	87,155,713	0.72	18.07	12.50	1.45
*D. immitis* (1)	87,899,012	0.73	18.23	12.50	1.46
*P. falciparum* (1)	23,326,872	0.19	4.84	12.50	0.39
Pool 5					
*B. malayi* (3)	261,467,139	2.10	52.49	12.50	4.20
*D. immitis* (3)	263,697,036	2.12	52.94	12.50	4.24
*P. falciparum* (3)	69,980,616	0.56	14.05	12.50	1.12

#### DNA sequencing

Before sequencing, quality control analysis was done to determine sample concentration using Qubit 4 fluorometer—Qubit 1x dsDNA High Sensitivity (HS) Assay kit (ThermoFisher; Q33231). Sequencing libraries were prepared using the Genomic DNA by Ligation Kit (Oxford Nanopore Technologies; SQK-LSK110). Due to differences in the fragment-length distributions of samples, the protocol was altered for some samples to ensure maximum library recovery. This involved using 1× AMPure for the clean-up of the AMX F adapter ligation reaction and using SFB for the washes during this clean-up reaction. Each library was run over one MinION flow cell (Oxford Nanopore Technologies; FLO-MIN106 R9.4.1) on the GridION X5 mk1. To maximise sequencing data yields, flow cells were treated with a nuclease flush and reload, using the Flow Cell Wash Kit (Oxford Nanopore Technologies; EXP-WSH004). ONT MinKNOW software (version 21.11.7) was used for the sequencing runs that originated fast5 files with read information. Guppy software (version 5.1.13) built in MinKNOW was used for basecalling in the high-accuracy setting, to convert fast5 to fastq files. The Deepseq team (University of Nottingham) performed quality control of the DNA sent for each pool. Qubit equipment calculated DNA concentrations of 9.28 ng/$\upmu$L, 12.1 ng/$\upmu$L, 32.8 ng/$\upmu$L, 29.2 ng/$\upmu$L and 24.2 ng/$\upmu$L for pools 1–5, respectively. The amount used for library preparation was approximately 1500 ng for all samples and the final amount loaded in each flow cell was 171.4 ng, 138.6 ng, 97.2 ng, 58.8 ng and 48.9 ng from each pool, after library preparation.

### Bioinformatic analysis

All analyses were performed on a Mac computer, running Monterey OS (3.6 GHz 10-core Intel Core i9 with memory 64GB 2667 MHz DDR4). The sequencing results were provided by the DeepSeq team in fastq format.

#### Concatenation of fastq files and quality control analysis

All fastq files from each experiment were concatenated using the cat function in the command line, e.g.: cat *.fq > output.fq

Nanoplot software (version 1.40.0) was used for quality control of sequencing reads.

#### Reference genomes and target regions for alignment

##### Whole genome analysis

The reference genomes for all species were downloaded from NCBI: *Ae. aegypti* LVP_AGWG strain GCF_002204515.2, *An. arabiensis* isolate DONGOLA GCF_016920 715.1, *An. coluzzii* (no isolate information) GCF_ 943734685.1, *B. malayi* FR3 strain GCF_000002995.4, *Cx. quinquefasciatus* JHB strain

GCF_015732765.1, *D. immitis* North_Portugal isolate GCA_024305405.1 (GenBank reference, no RefSeq genome available) and *P. falciparum* 3D7 strain GCF_000002765.5. For the control reference, the accession number of the 2 added species were: *An. gambiae* PEST strain (GCF_000005575.2) and *E. asinus* Dezhou breed (GCF_016077325.2). These species were added with the objective of adding a closely related species and a non-closely related species to the pool. According to each pool’s composition, the reference genomes were concatenated together using the cat function in the command line.

##### Target loci analysis

To find the exact sequences to use as reference, primers used in previous studies were mapped to the target loci belonging to each species and the sequence between them was retrieved using Benchling software. NCBI database was used to search for all sequences of each locus belonging to each of the vectors and pathogens used in pools 1–5 (Tables 5, 6, 7). Each sequence was saved as a different fasta file. All sequences were concatenated in the command line, using the cat function, according to the combinations chosen, originating a single reference per pool. WGS reads from the 5 different pools were mapped to each specific reference created using CLTq20prim.

**Table 5:** NCBI acession number of loci chosen to find the region of interest.

Species	NCBI ID
COX1	
*Ae. aegypti*	Reference sequence: NC_035159.1
*An. arabiensis*	Reference sequence: NC_028212.1
*An. coluzzii*	Reference sequence: NC_064604.1
*B. malayi*	Reference sequence: NC_004298.1
*Cx. quinquefasciatus*	Reference sequence: NC_014574.1
*D. immitis*	Reference sequence: NC_005305.1
ITS2	
*Ae. aegypti*	GenBank: GU980956.1
*An. arabiensis*	GenBank: DQ287773.1
*An. coluzzii*	GenBank: OL895513.1
*Cx. quinquefasciatus*	GenBank: GU562872.1
SINEs200	
*An. arabiensis*	GenBank: EU881887.1
*An. coluzzii*	GenBank: EU881868.1
IGS	
*An. arabiensis*	GenBank: AF470111.1
*An. coluzzii*	GenBank: KT284724.1
18srRNA	
*P. falciparum*	GenBank: M19172.1

**Table 6:** Primer sequences chosen to find target regions to use as reference.

Locus	Species	Forward primer (5’–3’)	Reverse primer (5’–3’)
Identification and abundance estimation			
COX1	All vectors	GGATTTGGAAATTGATTAGTTCCTT	AAAAATTTTAATTCCAGTTGGAACAGC^30^
COX1	*B. malayi* + *D. immitis*	TGATTGGTGGTTTTGGTAA	ATAAGTACGAGTATCAATATC^31^
ITS2	All vectors	TGTGAACTGCAGGACACATGAA	ATGCTTAAATTTAGGGGGTAGTC^32^
SINEs200	*Anopheles spp.*	TCGCCTTAGACCTTGCGTTA	CGCTTCAAGAATTCGAGATAC^33^
IGS	*Anopheles spp.*	GTGAAGCTTGGTGCGTGCT	GCACGCCGACAAGCTCA^30^
18srRNA	*P. falciparum*	GTTAAGGGAGTGAAGACGATCAGA	AACCCAAAGACTTTGATTTCTCATAA^34^

**Table 7:** NCBI IDs for each added species reference sequences.

Species	NCBI ID
COX1	
*An. funestus*	Reference sequence: NC_064603.1
*An. gambiae*	Reference sequence: NC_002084.1
ITS2	
*An. funestus*	GenBank: JN994136.1
*An. gambiae*	GenBank: EU104646.1
SINEs200	
*An. gambiae*	GenBank: EU881875.1
**IGS**	
*An. gambiae*	GenBank: AF470116.1
18srRNA	
*P. vivax*	GenBank: X13926.1

#### Read mapping using Minimap2 and Samtools

Minimap2 alignment software (version 2.24r1122) was used following the recommended for Nanopore sequencing^35^ and Samtools (version 1.16.1) was used for post alignment processing (the combination is here called CLT). Samtools view was used to convert the alignment files using the options to read headers (-h) and output bam files (-b). Samtools sort was used to sort the alignments in the bam file according to the reference genome provided. Samtools coverage was used to produce a text file report. Two extra pipelines were used by filtering reads after alignment for mapping quality (-q) superior to 20 and for mapping quality superior to 20 filtering for primary reads only (-F 0x100 and 0x800). The 2 pipelines with additional filters were named CLTq20 and CLTq20prim, respectively. Samtools flagstats was used to assess the number of mapped reads. When mapping reads to a control reference genome composed by the 7 different species mentioned above; the alignment was done using CLTq20prim and by adding the option -I 64g (required when using large references > 4Gb).

Reads that mapped to the different combo reference control were extracted (from the originated bam file from CLTq20prim pipeline) and converted back to fastq format using bedtools software (version 2.28.0). The reads were again mapped against a control reference genome to test if a possible amplicon approach would result in reads mapped to species absent in the pools. For this reason, COX1, ITS2, SINEs200 and IGS from *An. gambiae*, COX1 and ITS2 from *An. funestus* and 18srRNA from *P. vivax* were added to the combo 9 reference. This reference contained the combination of all target regions used for all species present in pools 4 and 5. The mapping was done as described previously for the CLTq20prim pipeline.

#### Read mapping using Epi2me agent

Two workflows were used for read mapping using the ONT software Epi2me Desktop agent (version 3.5.7). The first step consisted in uploading the fasta file containing the reference genomes using the Fasta Reference Upload workflow (version v2022.05.20-14092). Secondly, the Fastq Custom Alignment workflow (version v2021.11.26) was used to upload the merged fastq reads and align them to the reference.

#### Taxonomic classification using centrifuge

Reference genomes for taxonomic classification were provided as TaxIDs from the NCBI taxonomy as well as the database within (Refseq or Genbank): *Ae. aegypti*: 7159 Refseq, *An. arabiensis*: 7173 Refseq, *An. coluzzii*: 1518534 Refseq, *Cx. quinquefasciatus*: 7176 Refseq, *B. malayi* 6279 Refseq; *D. immitis*: 6287 Genbank and *P. falciparum*: 36329 Refseq. Centrifuge (version 1.0.4) was installed using conda. The analysis was done in the command line according to the manual instructions (assessed in https://ccb.jhu.edu/software/Centrifuge/manual.sht ml) by first downloading the NCBI taxonomy. The reference genomes were downloaded from the NCBI taxonomy to a folder (-o) by providing their TaxIDs (-t) (and specifying if used Refseq or Genbank) as well as each species class (-d). All individual genomes were concatenated into a single file in fasta format. Reference sequences were indexed with Centrifuge-build as specified by the manual, using 8 threads (for large genomes). Centrifuge was finally executed by providing the built index and fastq concatenated reads from a single pool.

#### Estimation of species abundance

Species abundance in the pools was calculated and defined as RA (RA). The number of mapped reads to chromosomes or contigs from the same species were summed up followed by division by the species’ genome length (to correct for different genome length sizes). The calculated values for all species were added up (total abundance) and the RA for each species calculated as a ratio in relation to the total abundance. The values were converted so the total abundance in each pool was equal to 1 (RA).

#### Statistical analysis

##### Correlation between estimated species abundance and sample composition

To evaluate the performance of the sequencing technology in identifying vector and pathogens in relation to the different pools content, the correlation between the species’ ratio in the samples prepared and the abundance values obtained from the sequencing experiments were measured. As each pool contained a small number of species (4 in pools 1–3 and 7 in pools 4 and 5), the statistical analysis was done by combining all values per species from each pipeline used. Shapiro-Wilk was used to test if the data followed a normal distribution to help infer the best correlation test to use. As samples did not follow a normal distribution (P-value < 0.05), the non-parametric Spearman’s rank correlation test was used. Both tests were performed using *R* software (version 2022.12.0+353) using the readxl, psych, tidyverse and corrplot packages (versions 4.2.0). As multiple P-values were produced in this test, the Bonferroni correction method was used to adjust for a statistical type I error.

##### Comparison of Spearman’s rank correlation values between different correlation tests

When comparing P-values across different correlation tests, the Fisher’s Z-test was used. It measures if the correlation between two datasets differs (and its significance) between two independent studies. The test was done using *R* software (version 2022.12.0+353) using the diffcor package (version 4.2.0).

##### Comparison between DNA content for each vector and read correction

Twelve DNA extractions were performed on individual mosquitoes from each species to determine the DNA concentration after extraction. Shapiro-Wilk test was used to test if the different DNA concentrations from each vector followed a normal distribution, followed by the Barlett’s test to assess homogeneity of variances. Kruskall-Wallis assessed if the means were significantly different between all datasets. Dunn’s test was used to analyse if the average DNA concentration was significantly different between each species. Bonferroni method was used for P-value correction when performing multiple comparisons. For correction (for DNA concentration), RA of each species was divided by the mean DNA concentration obtained for that species. The final RA values were obtained by dividing each individual species corrected value by the sum of all values after the correction to obtain a total final abundance of 1. Only the values of RA obtained by the software with the highest correlation to the pools’ true composition were corrected. Parasite gDNA was supplied at 50 ng/$\upmu$L and further diluted 4 times before being added to the pools. For this reason, all RA values for *B. malayi*, *D. immitis* and *P. falciparum* were corrected, assuming a concentration of 12.5 ng/$\upmu$L. All analysis were performed using *R* software (version 2022.12.0+353) using readxl, car, stats, psych, and FSA packages (all version 4.2.0). The Spearman’s correlation was used to measure the association between corrected values and the pools’ composition. Correlation values before and after correction for DNA concentration were compared using Fisher’s Z-test to determine if the difference was statistically significant. All analysis were performed using *R* software (version 2022.12.0+353) using the readxl, psych and diffcor packages (all version 4.2.0).

*Sequencing coverage and depth*

Samtools coverage software was used to determine the values of sequencing coverage and depth from the CLTq20prim pipeline output (Epi2me Agent values of sequencing depth and coverage were detailed in the platform as plots for each region).

## Supplementary Information

Supplementary Information.

## Data Availability

The data analysis performed is available in the Supplementary materials section. The sequencing data can be accessed in the NIH BioProject PRJNA1404320 (Pools 1–5; SRA numbers SRR36902432–SRR36902436). All the generated data is available in the Supplementary materials section. Raw data is available upon request. The sequencing data can be accessed in the NIH BioProject PRJNA1404320 (Pools 1–5; SRA numbers SRR36902432–SRR36902436).
