# Supplementary material for: In silico DNA barcoding surpasses whole genome sequencing for species identification from vector surveillance pools
Source: Sci Rep. 2026 Feb 23;16:10231. doi: 10.1038/s41598-026-39937-y (PMC13031962; doi:10.1038/s41598-026-39937-y)
Supplement: Supplementary file 1 — Supplementary Information. [file 41598_2026_39937_MOESM1_ESM.pdf]

In silico DNA barcoding surpasses whole genome sequencing for species identification from vector surveillance pools

Supplementary Materials

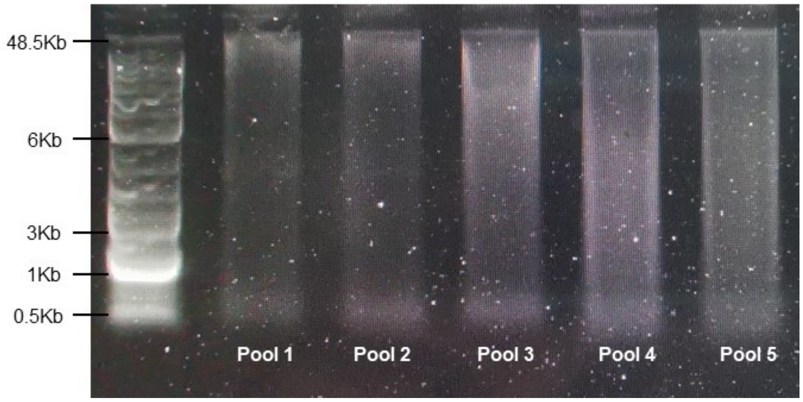

Figure S1. **Gel electrophoresis of pooled samples (1-5) for determination of DNA fragment length.**
DNA extractions from pooled mosquitoes, in 0.7% agarose gel. Quick-LoadPurple Extended 1 kb Plus DNA Ladder NEB. Samples subjected to current for 2h 100V.

Table S1. Mapped reads to species individual chromosomes using CLT

	Number of mapped reads						
**Species**	**Chr 1**	**Chr 2**	**Chr 3**	**Chr X**	**Mitochondrial Chr**	**Undefined**	**Total**
				**Pool 1**			
Ae. aegypti	105932	162324	141911	-	166	18062	428395
An. arabiensis	-	815893	757577	43317	31357	70445	1718589
An. coluzzii	-	2235567	1900857	667417	21332	531590	5356763
Cx. quinquefasciatus	25638	44496	39701	-	618	5	110458
				**Pool 2**			
Ae. aegypti	196130	301521	261961	-	287	32903	792802
An. arabiensis	-	722268	670374	41364	29989	65250	1529245
An. coluzzii	-	1936198	1648901	590723	15957	457026	4648805
Cx. quinquefasciatus	48402	82134	74393	-	1226	13	206168
				**Pool 3**			
Ae. aegypti	356713	553317	477084	-	244	57480	1444838
An. arabiensis	-	701001	661333	45351	18754	79810	1506249
An. coluzzii	-	1839471	1584678	518135	10474	441006	4393764
Cx. quinquefasciatus	208657	348913	327084	-	2115	45	886814
				**Pool 4**			
Ae. aegypti	655603	976108	851527	-	671	108865	2592774
An. arabiensis	-	709124	605621	85714	7049	148661	1556169
An. coluzzii	-	725333	627221	201479	4293	152046	1710372
B.malayi	-	-	-	-	-	19939	19939
Cx. quinquefasciatus	1412592	2386656	2248807	-	7813	369	6056237
D.immitis	-	-	-	-	-	4376	4376
P.falciparum	-	-	-	-	-	249	249
				**Pool 5**			
Ae. aegypti	551018	832852	723594	-	385	87273	219522
An. arabiensis	-	482879	421985	57920	6260	113714	1082758
An. coluzzii	-	512548	451243	143104	3705	109328	1219928
B.malayi	-	-	-	-	-	32201	32201
Cx. quinquefasciatus	1188104	2021193	1904514	-	6665	304	5120780
D.immitis	-	-	-	-	-	8110	8110
P.falciparum	-	-	-	-	-	382	382

Table S2. **Mapped reads to species individual chromosomes using CLTq20**

	Number of mapped reads						
**Species**	**Chr 1**	**Chr 2**	**Chr 3**	**Chr X**	**Mitochondrial Chr**	**Undefined**	**Total**
				**Pool 1**			
Ae. aegypti	61821	100475	85657	-	9	4761	2527723
An. arabiensis	-	472584	450925	13395	12400	4079	953383
An. coluzzii	-	1723129	1426296	567613	4625	49401	3771064
Cx. quinquefasciatus	14935	25372	23496	-	534	5	64342
				**Pool 2**			
Ae. aegypti	113774	187252	158669	-	8	8983	468686
An. arabiensis	-	413601	394563	13653	12523	3540	837880
An. coluzzii	-	1478594	1226124	495081	3040	42085	3244924
Cx. quinquefasciatus	28490	47029	43611	-	1061	11	120202
				**Pool 3**			
Ae. aegypti	207768	342553	287743	-	9	16717	854790
An. arabiensis	-	435747	417928	19054	8801	5448	886978
An. coluzzii	-	1448654	1215930	445138	2312	42122	3154156
Cx. quinquefasciatus	123928	210040	195263	-	1929	33	531193
				**Pool 4**			
Ae. aegypti	375796	615389	523322	-	11	23561	1538079
An. arabiensis	-	450957	393171	61082	2362	6797	914369
An. coluzzii	-	456765	394101	163838	717	8118	1023539
B.malayi	-	-	-	-	-	16204	16204
Cx. quinquefasciatus	882710	1531851	1418975	-	7033	321	3840890
D.immitis	-	-	-	-	-	3043	3043
P.falciparum	-	-	-	-	-	60	60
				**Pool 5**			
Ae. aegypti	325077	529482	450837	-	4	22688	1328088
An. arabiensis	-	342919	302143	43165	2758	6836	697821
An. coluzzii	-	362226	315904	119940	857	8626	807553
B.malayi	-	-	-	-	-	26905	26905
Cx. quinquefasciatus	744957	1296088	1204687	-	6161	264	3252157
D.immitis	-	-	-	-	-	6583	6583
P.falciparum	-	-	-	-	-	154	154

Table S3. **Mapped reads to species individual chromosomes using CLTq20prim**

	Number of mapped reads						
**Species**	**Chr 1**	**Chr 2**	**Chr 3**	**Chr X**	**Mitochondrial Chr**	**Undefined**	**Total**
				**Pool 1**			
Ae. aegypti	45749	75625	64532	-	5	3382	189293
An. arabiensis	-	437019	416448	8821	11783	3193	877264
An. coluzzii	-	1610496	1326379	525045	3850	45560	3511330
Cx. quinquefasciatus	11561	19186	17857	-	394	3	49001
				**Pool 2**			
Ae. aegypti	83766	139084	118224	-	6	6374	347454
An. arabiensis	-	379057	361355	9537	11692	2755	764396
An. coluzzii	-	1372075	1131218	454017	2517	38268	2998095
Cx. quinquefasciatus	21825	35279	32721	-	770	9	90604
				**Pool 3**			
Ae. aegypti	153116	256315	213917	-	9	12043	635400
An. arabiensis	-	400740	384084	14130	8306	4397	811657
An. coluzzii	-	1349018	1126193	409038	1907	38383	2924539
Cx. quinquefasciatus	89912	151975	142917	-	1351	19	386174
				**Pool 4**			
Ae. aegypti	332532	544729	463291	-	8	20513	1361073
An. arabiensis	-	428872	372983	57385	2255	6215	867710
An. coluzzii	-	433884	373269	154735	620	770	970208
B.malayi	-	-	-	-	-	14281	14281
Cx. quinquefasciatus	787676	1372007	1271859	-	5945	276	3437763
D.immitis	-	-	-	-	-	2788	2788
P.falciparum	-	-	-	-	-	36	36
				**Pool 5**			
Ae. aegypti	138798	226303	192994	-	0	9593	567688
An. arabiensis	-	162458	143584	20062	1353	3168	330625
An. coluzzii	-	171580	149004	56315	359	4209	381467
B.malayi	-	-	-	-	-	12000	12000
Cx. quinquefasciatus	319684	561135	522659	-	2647	104	1406229
D.immitis	-	-	-	-	-	3049	3049
P.falciparum	-	-	-	-	-	48	48

Table S4. **Mapped reads to species individual chromosomes using Epi2me Agent**

	Number of mapped reads						
**Species**	**Chr 1**	**Chr 2**	**Chr 3**	**Chr X**	**Mitochondrial Chr**	**Undefined**	**Total**
			**Pool 1**				
Ae. aegypti	67188	103459	90915	-	114	11727	273403
An. arabiensis	-	764569	691652	28201	28509	92180	1605111
An. coluzzii	-	1985846	1685066	592191	16501	497237	4776841
Cx. quinquefasciatus	17551	29991	26842	-	454	4	74842
			**Pool 2**				
Ae. aegypti	123126	189690	165822	-	211	21198	500047
An. arabiensis	-	665783	602958	27310	25956	58451	1380458
An. coluzzii	-	1702115	1445371	517531	11841	422530	4099388
Cx. quinquefasciatus	32726	54522	49194	-	885	10	137337
			**Pool 3**				
Ae. aegypti	21875	343585	294622	-	226	63663	920851
An. arabiensis	-	651874	599736	29795	17157	45330	1343892
An. coluzzii	-	1616331	1390315	454476	7584	410245	3878951
Cx. quinquefasciatus	125971	209317	198058	-	1515	24	534885
			**Pool 4**				
Ae. aegypti	540958	801355	699411	-	550	97527	2139801
An. arabiensis	-	663061	563618	78264	6499	142203	1453645
An. coluzzii	-	674459	581706	185914	3563	136754	1582396
B.malayi	-	-	-	-	-	17294	17294
Cx. quinquefasciatus	1173790	1992580	1977580	-	6624	12	5050586
D.immitis	-	-	-	-	-	3894	3894
P.falciparum	-	-	-	-	-	102	102
			**Pool 5**				
Ae. aegypti	422045	635058	553232	-	305	66852	1677492
An. arabiensis	-	448645	389478	51719	5931	108627	1004400
An. coluzzii	-	471360	414083	130166	2993	103364	1121966
B.malayi	-	-	-	-	-	28379	28379
Cx. quinquefasciatus	918420	1574482	1483454	-	5672	231	3982259
D.immitis	-	-	-	-	-	7343	7343
P.falciparum	-	-	-	-	-	168	168

Table S5. **Classified reads to species using Centrifuge**

Species	Read number	Unique read number
**Pool 1**		
Ae. aegypti	333637	288320
An. arabiensis	2349294	1929441
An. coluzzii	4596297	4196524
Cx. quinquefasciatus	600044	261892
**Pool 2**		
Ae. aegypti	591298	519333
An. arabiensis	2129728	1703181
An. coluzzii	3964552	3600674
Cx. quinquefasciatus	866192	415041
**Pool 3**		
Ae. aegypti	960129	887641
An. arabiensis	1843612	1618903
An. coluzzii	3704163	3436075
Cx. quinquefasciatus	648841	571595
**Pool 4**		
Ae. aegypti	2461755	2043539
An. arabiensis	1831406	1446947
An. coluzzii	1684545	1351648
B.malayi	24622	20069
Cx. quinquefasciatus	5661889	5017438
D.immitis	32914	14600
P.falciparum	383	59
**Pool 5**		
Ae. aegypti	1846576	1633678
An. arabiensis	1210322	1035423
An. coluzzii	1140208	990866
B.malayi	33887	30311
Cx. quinquefasciatus	4306379	3988363
D.immitis	31042	15426
P.falciparum	290	36

Table S6. Sequencing depth and coverage per species from pools 1-5

	Sequencing coverage and (/) depth					
**Species**	**Chr 1**	**Chr 2**	**Chr 3**	**Chr X**	**Mitochondrial Chr**	**Unplaced**
			**Pool 1**			
Ae. aegypti	19.07/0.23	20.37/0.25	20.24/0.24	-	16.69/0.20	12.16/0.17
An. arabiensis	-	68.04/4.34	71.92/5.32	17.00/0.35	100.00/1184.60	6.88/0.17
An. coluzzii	-	96.51/23.29	96.17/21.46	97.32/27.37	100.00/201.07	37.26/3.64
Cx. quinquefasciatus	10.12/0.12	9.93/0.11	10.09/0.11	-	100.00/29.32	20.53/0.26
			**Pool 2**			
Ae. aegypti	30.00/0.41	32.52/0.45	32.45/0.45	-	27.74/0.31	17.22/0.26
An. arabiensis	-	68.92/3.79	72.68/4.59	22.41/0.39	100.00/1191.33	8.07/0.16
An. coluzzii	-	96.19/19.21	95.85/17.68	97.11/22.84	98.23/133.76	35.68/3.19
Cx. quinquefasciatus	18.54/0.22	17.45/0.21	17.27/0.21	-	100.00/65.74	8.81/0.09
			**Pool 3**			
Ae. aegypti	44.32/0.77	47.79/0.85	46.86/0.81	-	63.24/1.10	21.17/0.38
An. arabiensis	-	80.29/4.63	82.21/5.51	39.10/0.67	100.00/1011.76	11.94/0.30
An. coluzzii	-	96.01/19.85	95.40/18.72	97.00/22.56	99.94/115.98	34.49/3.05
Cx. quinquefasciatus	51.79/1.02	51.71/1.00	54.74/1.07	-	100.00/136.61	19.50/0.31
			**Pool 4**			
Ae. aegypti	38.53/0.59	40.75/0.63	40.20/0.62	-	18.35/0.22	14.55/0.23
An. arabiensis	-	75.68/1.88	75.61/1.90	57.74/0.97	100.00/152.95	11.22/0.25
An. coluzzii	-	74.92/1.97	74.36/1.95	85.73/2.68	95.88/28.54	14.70/0.36
B. malayi	-	-	-	-	-	16.52/0.26
Cx. quinquefasciatus	75.55/2.99	76.88/3.10	78.60/3.24	-	100.00/264.14	21.34/1.02
D. immitis	-	-	-	-	-	5.01/0.06
P. falciparum	-	-	-	-	-	0.49/0.00
			**Pool 5**			
Ae. aegypti	26.95/0.35	28.63/0.37	28.32/0.37	-	0.00/0.00	12.13/0.17
An. arabiensis	-	58.96/1.02	59.51/1.05	37.60/0.50	100.00/120.10	8.93/0.15
An. coluzzii	-	60.50/1.11	60.11/1.11	71.51/1.43	996.36/17.35	11.31/0.22
B. malayi	-	-	-	-	-	24.38/0.43
Cx. quinquefasciatus	67.48/1.73	69.24/1.79	71.04/1.89	-	100.00/166.54	17.80/0.39
D. immitis	-	-	-	-	-	8.72/0.09
P. falciparum	-	-	-	-	-	0.62/0.01

Table S7. **Mapped reads to control reference genome using CLTq20prim**

	Number of mapped reads						
**Species**	**Chr 1**	**Chr 2**	**Chr 3**	**Chr X**	**Mitochondrial Chr**	**Undefined**	**Total**
**Pool 1**							
Ae. aegypti	45652	75521	64477	-	5	3352	189007
An. arabiensis	-	519409	201068	4957	2845	2892	731171
An. coluzzii	-	682076	611514	180650	2635	15643	1492518
An. Gambian	-	535260	527960	130500	2015	130816	1326551
B.malayi	-	-	-	-	-	4	4
Cx. quinquefasciatus	11524	19114	17791	-	393	3	48825
D.immitis	-	-	-	-	-	191	191
E.asinus	-	-	-	-	-	405	405
P.falciparum	-	-	-	-	-	0	0
**Pool 2**							
Ae. aegypti	83625	138905	118069	-	6	6307	346912
An. arabiensis	-	452366	177315	5966	2396	2592	640635
An. coluzzii	-	577722	521057	155658	2023	12854	1269314
An. Gambian	-	452143	447114	111353	1524	111496	1123630
B.malayi	-	-	-	-	-	23	23
Cx. quinquefasciatus	21752	35171	32619	-	766	9	90317
D.immitis	-	-	-	-	-	268	268
E.asinus	-	-	-	-	-	605	605
P.falciparum	-	-	-	-	-	0	0
**Pool 3**							
Ae. aegypti	152805	256036	213631	-	8	11949	670276
An. arabiensis	-	481023	204858	11092	1674	4087	714256
An. coluzzii	-	580282	529489	150829	1549	14321	1276470
An. Gambian	-	450427	455313	103437	1172	110188	1120537
B.malayi	-	-	-	-	-	28	28
Cx. quinquefasciatus	89700	151617	142472	-	1347	18	385154
D.immitis	-	-	-	-	-	80	80
E.asinus	-	-	-	-	-	62	62
P.falciparum	-	-	-	-	-	0	0
**Pool 4**							
Ae. aegypti	332197	544371	462960	-	8	20447	1359983
An. arabiensis	-	433099	271777	55414	454	5227	765971
An. coluzzii	-	159327	158134	41510	471	1899	361341
An. Gambian	-	134902	141298	30641	282	32141	339264
B.malayi	-	-	-	-	-	14262	14262
Cx. quinquefasciatus	786425	1369671	1269591	-	5938	276	3431901
D.immitis	-	-	-	-	-	3023	3023
E.asinus	-	-	-	-	-	27	27
P.falciparum	-	-	-	-	-	35	35
**Pool 5**							
Ae. aegypti	276883	451237	384831	-	3	18949	1131903
An. arabiensis	-	332839	214413	38991	679	5257	592179
An. coluzzii	-	137392	136976	37196	582	277007	314203
An. Gambian	-	115510	121434	26770	387	26126	290227
B.malayi	-	-	-	-	-	24082	24082
Cx. quinquefasciatus	638994	1119903	1042619	-	5192	216	2806924

Continued on next page

Table S7 – *Continued from previous page*

**Species**	**Chr 1**	**Chr 2**	**Chr 3**	**Chr X**	**Mitochondrial Chr**	**Undefined**	**Total**
D.immitis	-	-	-	-	-	6479	6479
E.asinus	-	-	-	-	-	24	24
P.falciparum	-	-	-	-	-	107	107

Table S8. **Classified reads to species with Centrifuge when using a control reference genome**

Species	Read number	Unique read number
	**Pool 1**	
Ae. aegypti	333637	288320
An. arabiensis	2349294	1929441
An. coluzzii	4596297	4196524
An. Gambian	600044	261892
B.malayi	2349294	1929441
Cx. quinquefasciatus	4596297	4196524
D.immitis	600044	261892
E.asinus	4596297	4196524
P.falciparum	600044	261892
	**Pool 2**	
emphAe. aegypti	333637	288320
An. arabiensis	2349294	1929441
An. coluzzii	4596297	4196524
An. Gambian	600044	261892
B.malayi	2349294	1929441
Cx. quinquefasciatus	4596297	4196524
D.immitis	600044	261892
E.asinus	4596297	4196524
P.falciparum	600044	261892
	**Pool 3**	
Ae. aegypti	333637	288320
An. arabiensis	2349294	1929441
An. coluzzii	4596297	4196524
An. Gambian	600044	261892
B.malayi	2349294	1929441
Cx. quinquefasciatus	4596297	4196524
D.immitis	600044	261892
E.asinus	4596297	4196524
P.falciparum	600044	261892
	**Pool 4**	
emphAe. aegypti	333637	288320
An. arabiensis	2349294	1929441
An. coluzzii	4596297	4196524
An. Gambian	600044	261892
B.malayi	2349294	1929441
Cx. quinquefasciatus	4596297	4196524
D.immitis	600044	261892
E.asinus	4596297	4196524
P.falciparum	600044	261892
	**Pool 5**	
emphAe. aegypti	333637	288320
An. arabiensis	2349294	1929441
An. coluzzii	4596297	4196524
An. Gambian	600044	261892
B.malayi	2349294	1929441
Cx. quinquefasciatus	4596297	4196524
D.immitis	600044	261892
E.asinus	4596297	4196524
P.falciparum	600044	261892

Table S9. **Sequencing depth and coverage per species, from pools 1-5, when mapping reads to a control reference genome**

	Sequencing coverage (%) and (/) depth (fold)					
**Species**	**Chr 1**	**Chr 2**	**Chr 3**	**Chr X**	**Mitochondrial Chr**	**Unplaced**
			**Pool 1**			
Ae. aegypti	19.06/0.23	20.35/0.25	20.23/0.24	-	16.69/0.20	12.62/0.17
An. arabiensis	-	56.04/6.67	56.85/2.58	14.33/0.22	98.59/207.60	4.51/0.21
An. coluzzii	-	78.74/9.56	92.89/11.14	92.74/12.08	100.00/144.04	22.28/1.49
An. gambiae	-	71.44/7.50	83.04/8.69	84.78/7.74	100.00/144.04	62.04/5.37
B. malayi	-	-	-	-	-	1.47/0.01
Cx. quinquefasciatus	10.42/0.12	9.92/0.11	10.08/0.11	-	100.00/29.31	20.53/0.26
D. immitis	-	-	-	-	-	0.03/0.00
E. asinus	-	-	-	-	-	0.01/0.00
P. falciparum	-	-	-	-	-	-
			**Pool 2**			
Ae. aegypti	29.99/0.41	32.48/0.45	32.42/0.45	-	27.74/0.31	17.34/0.26
An. arabiensis	-	58.29/5.72	58.32/2.31	20.08/0.29	99.62/181.12	6.10/0.19
An. coluzzii	-	77.14/7.84	91.68/9.18	91.79/9.99	100.00/110.42	21.69/1.30
An. gambiae	-	71.44/7.50	83.04/8.69	83.19/6.47	95.41/92.2	60.61/4.67
B. malayi	-	-	-	-	-	4.78/0.13
Cx. quinquefasciatus	18.51/0.22	17.41/0.21	17.25/0.21	-	100.00/65.35	8.81/0.09
D. immitis	-	-	-	-	-	0.13/0.00
E. asinus	-	-	-	-	-	0.01/0.00
P. falciparum	-	-	-	-	-	-
			**Pool 3**			
Ae. aegypti	44.29/0.77	47.77/0.85	46.83/0.81	-	60.85/1.06	21.45/0.38
An. arabiensis	-	73.71/6.53	72.54/3.01	37.39/0.56	98.35/141.72	9.89/0.33
An. coluzzii	-	76.33/8.11	90.93/9.68	90.44/9.77	100.00/93.92	20.13/1.38
An. gambiae	-	71.44/7.50	83.04/8.69	100.00/93.92	94.50/72.83	62.41/5.84
B. malayi	-	-	-	-	-	0.37/0.00
Cx. quinquefasciatus	51.68/1.02	51.66/1.00	54.72/1.07	-	100.00/136.02	23.27/0.37
D. immitis	-	-	-	-	-	0.38/0.00
E. asinus	-	-	-	-	-	0.00/0.00
P. falciparum	-	-	-	-	-	-
			**Pool 4**			
Ae. aegypti	38.51/0.59	40.73/0.63	40.18/0.62	-	18.35/0.22	14.53/0.23
An. arabiensis	-	72.80/1.95	66.03/1.45	57.02/0.95	91.75/28.26	9.89/0.21
An. coluzzii	-	38.04/0.72	49.49/0.94	46.51/0.90	98.54/22.66	6.18/0.14
An. gambiae	-	35.82/0.66	43.96/0.82	39.31/0.70	87.53/14.99	40.74/1.00
B. malayi	-	-	-	-	-	16.28/0.26
Cx. quinquefasciatus	75.50/2.98	76.82/3.09	78.57/3.24	-	100.00/263.89	21.34/1.02
D. immitis	-	-	-	-	-	8.84/0.10
E. asinus	-	-	-	-	-	0.00/0.00
P. falciparum	-	-	-	-	-	0.49/0.00

Continued on next page

Table S9 – *Continued from previous page*

**Species**	**Chr 1**	**Chr 2**	**Chr 3**	**Chr X**	**Mitochondrial Chr**	**Unplaced**
			**Pool 5**			
Ae. aegypti	42.88/0.70	45.13/0.74	44.57/0.74	-	12.65/0.16	17.02/0.29
An. arabiensis	-	76.03/2.15	69.74/1.63	57.72/0.97	87.24/44.95	11.58/0.26
An. coluzzii	-	43.57/0.87	54.76/1.13	52.45/1.10	99.67/29.82	8.96/0.50
An. gambiae	-	79.10/1.57	48.63/0.98	43.57/0.84	93.53/21.32	45.69/1.07
B. malayi	-	-	-	-	-	29.64/0.66
Cx. quinquefasciatus	78.54/3.45	79.41/3.58	81.00/3.78	-	100.00/325.17	21.05/1.01
D. immitis	-	-	-	-	-	19.67/0.30
E. asinus	-	-	-	-	-	0.00/0.00
P. falciparum	-	-	-	-	-	1.46/0.01

Table S10. Number of mapped reads per species to target identification loci

Target loci	*Ae. aegypti*	*An. arabiensis*	*An. coluzzii*	*B.malayi*	*Cx. quinquefasciatus*	*D.immitis*	*P.falciparum*
				**Pool 1**			
COX1	192	1026	404	-	38	-	-
COX1	443	70	28336	-	29	-	-
Combo 0	635	1096	28737	-	657	-	-
Combo 1	192	1080	27829	-	38	-	-
Combo 2	635	1096	72034	-	67	-	-
Combo 3	635	1142	46690	-	67	-	-
Combo 4	635	1142	89977	-	67	-	-
				**Pool 2**			
COX1	340	1048	188	-	87	-	-
COX1	910	116	23726	-	70	-	-
Combo 0	1143	1163	23472	-	140	-	-
Combo 1	340	1140	55540	-	87	-	-
Combo 2	1143	1164	59964	-	140	-	-
Combo 3	1143	1238	39052	-	140	-	-
Combo 4	1191	3953	92115	-	144	-	-
				**Pool 3**			
COX1	523	789	213	-	192	-	-
COX1	1196	248	21942	-	279	-	-
Combo 0	1719	1037	22155	-	470	-	-
Combo 1	523	1030	5667	-	192	-	-
Combo 2	1719	1039	59549	-	470	-	-
Combo 3	1719	1268	38560	-	470	-	-
Combo 4	1719	1270	75956	-	470	-	-
				**Pool 4**			
COX1	639	140	82	22	638	3	0
COX1	1901	688	3619	0	2763	0	0
Combo 5	2540	828	3701	22	3401	3	0
Combo 6	639	661	8273	22	638	3	0
Combo 7	2540	830	9532	22	3401	3	0
Combo 8	2540	1343	6048	22	3401	3	0
Combo 9	2540	1344	11879	22	3401	3	0
				**Pool 5**			
COX1	476	166	76	32	599	2	-
COX1	1804	544	2966	-	2308	-	-
Combo 5	2280	709	3043	32	2907	2	0
Combo 6	476	667	8835	32	599	2	0
Combo 7	2280	713	9655	32	2907	2	0
Combo 8	2280	1200	5168	32	2907	2	0
Combo 9	2280	1204	11780	32	2907	2	0

Table S11. Sequencing coverage and depth when mapping WGS reads to individual target loci

		Sequencing coverage (%)/ and depth (fold -x)				
**Species**	**COX1**	**COX1**	**SINEs200**	**IGS**		
			**Pool 1**			
Ae. aegypti	100.00/91.34	100.00/391.23	-	-	*An. arabiensis*	100.00/676.66
100.00/57.94	0.00	100.00/44.91	*An. coluzzii*	100.00/225.04	100.00/23105.60	100.00/19455.40
100.00/17075.90	*Cx. quinquefasciatus*	100.00/26.30	100.00/22.49	-	-	
			**Pool 2**			
Ae. aegypti	100.00/183.53	100.00/817.56	-	-	*An. arabiensis*	100.00/748.47
100.00/99.40	100.00/1.00	100.00/83.70	*An. coluzzii*	100.00/104.91	100.00/19192.10	100.00/21246.70
100.00/15460.90	*Cx. quinquefasciatus*	100.00/61.95	100.00/60.37	-	-	
			**Pool 3**			
Ae. aegypti	100.00/271.63	100.00/1052.72	-	-	98.90/0.99	*An. arabiensis*
100.00/571.64	100.00/203.69	100.00/1.93	100.00/223.27	*An. coluzzii*	100.00/129.80	100.00/17973.60
100.00/16669.90	100.00/15681.30	*Cx. quinquefasciatus*	100.00/135.04	100.00/221.61	-	-
100.00/1.80						
			**Pool 4**			
Ae. aegypti	100.00/246.22	100.00/1367.92	-	-	*An. arabiensis*	100.00/73.64
100.00/397.59	90.78/1.69	100.00/479.75	*An. coluzzii*	100.00/44.27	100.00/2060.28	81.66/2383.73
100.00/2144.06	*B. malayi*	100.00/15.51	-	-	-	*Cx. quinquefasciatus*
100.00/320.27	100.00/1641.70	-	-	*D. immitis*	100.00/2.18	-
-	-					
			**Pool 5**			
Ae. aegypti	100.00/227.01	100.00/1443.06	-	-	*An. arabiensis*	100.00/91.82
100.00/385.37	100.00/3.73	100.00/465.38	*An. coluzzii*	100.00/41.47	100.00/1987.22	100.00/2813.45
100.00/1997.65	*B. malayi*	100.00/23.84	-	-	-	*Cx. quinquefasciatus*
100.00/355.10	100.00/1591.18	-	-	*D. immitis*	100.00/1.96	-
-	-					

Table S12. Number of mapped reads per species to control reference loci

Species	COX1	COX1	IGS	SINEs200	18srRNA
		**Pool 1**			
Ae. aegypti	382	886	-	-	-
An. arabiensis	206	140	92	0	-
An. coluzzii	628	28690	30	86596	-
An. funestus	0	0	-	-	-
An. gambiae	10	6	0	0	-
B. malayi	0	-	-	-	-
Cx. quinquefasciatus	76	58	-	-	-
D. immitis	0	-	-	-	-
P. falciparum	-	-	-	-	0
P. vivax	-	-	-	-	0
		**Pool 2**			
Ae. aegypti	680	1606	-	-	-
An. arabiensis	236	230	146	2	-
An. coluzzii	316	23646	36	72974	-
An. funestus	0	0	-	-	-
An. gambiae	2	0	0	0	-
B. malayi	0	-	-	-	-
Cx. quinquefasciatus	174	106	-	-	-
D. immitis	0	-	-	-	-
P. falciparum	-	-	-	-	0
P. vivax	-	-	-	-	0
		**Pool 3**			
Ae. aegypti	1046	2392	-	-	-
An. arabiensis	220	492	462	4	-
An. coluzzii	336	21704	42	74790	-
An. funestus	0	0	-	-	-
An. gambiae	0	0	2	0	-
B. malayi	0	-	-	-	-
Cx. quinquefasciatus	382	558	-	-	-
D. immitis	0	-	-	-	-
P. falciparum	-	-	-	-	0
P. vivax	-	-	-	-	0
		**Pool 4**			
Ae. aegypti	1278	3802	-	-	-
An. arabiensis	22	1366	1018	4	-
An. coluzzii	120	2882	8	11662	-
An. funestus	0	0	-	-	-
An. gambiae	2	0	0	0	-
B. malayi	44	-	-	-	-
Cx. quinquefasciatus	1276	5526	-	-	-
D. immitis	6	-	-	-	-
P. falciparum	-	-	-	-	0
P. vivax	-	-	-	-	0
		**Pool 5**			
Ae. aegypti	952	3608	-	-	-
An. arabiensis	22	1080	980	8	-
An. coluzzii	118	2726	10	13224	-
An. funestus	0	0	-	-	-
An. gambiae	2	0	0	0	-
B. malayi	64	-	-	-	-

Continued on next page

Table S12 – *Continued from previous page*

**Species**	**COX1**	**COX1**	**IGS**	**SINEs200**	**18srRNA**
Cx. quinquefasciatus	1198	4616	-	-	-
D. immitis	4	-	-	-	-
P. falciparum	-	-	-	-	0
P. vivax	-	-	-	-	0

Table S13. **Sequencing coverage and depth when mapping extracted WGS reads to control target loci for species identification and abundance estimation**

	Sequencing coverage (%)/ and depth (fold -x)			
**Species**	**COX1**	**COX1**	**SINEs200**	**IGS**
		**Pool 1**		
Ae. aegypti	100/182.39	100/782.46	-	-
An. arabiensis	100/191.56	100/115.87	100/0.00	100/89.81
An. coluzzii	100/355.90	100/24547.3	100/38910.80	100/24.39
An. gambiae	74.53/6.99	100/4.56	0.00/0.00	0.00/0.00
B. malayi	0.00/0.00	-	-	-
Cx. quinquefasciatus	100/52.59	100/44.95	-	-
D. immitis	0.00/0.00	-	-	-
		**Pool 2**		
Ae. aegypti	100/367.05	100/1445.01	-	-
An. arabiensis	100/213.76	100/196.85	100/2.00	100/141.69
An. coluzzii	100/175.95	100/20192.90	100/32728.70	100/31.06
An. gambiae	71.01/1.42	0.00/0.00	0.00/0.00	0.00/0.00
B. malayi	0.00/0.00	-	-	-
Cx. quinquefasciatus	100/123.90	100/88.36	-	-
D. immitis	0.00/0.00	-	-	-
		**Pool 3**		
Ae. aegypti	100/543.26	100/2105.45	-	-
An. arabiensis	100/209.14	100/405.20	100/3.85	100/446.53
An. coluzzii	100/202.65	100/18628.50	100/33339.40	100/37.64
An. gambiae	0.00/0.00	0.00/0.00	0.00/0.00	96.09/1.92
B. malayi	0.00/0.00	-	-	-
Cx. quinquefasciatus	100/268.64	100/443.22	-	-
D. immitis	0.00/0.00	-	-	-
		**Pool 4**		
Ae. aegypti	100/492.43	100/2735.84	-	-
An. arabiensis	100/18.66	100/790.09	98.78/3.38	100/950.61
An. coluzzii	100/69.64	100/1790.69	81.67/4767.45	100/6.69
An. gambiae	75.25/1.50	0.00/0.00	0.00/0.00	0.00/0.00
B. malayi	100/31.02	-	-	-
Cx. quinquefasciatus	100/640.55	100/3283.41	-	-
D. immitis	99.85/4.36	-	-	-
		**Pool 5**		
Ae. aegypti	100/454.02	100/2886.12	-	-
An. arabiensis	100/18.61	100/766.62	100/7.46	100/928.81
An. coluzzii	99.85/64.22	100/1979.90	100/5626.90	100/8.29
An. gambiae	67.20/1.34	0.00/0.00	0.00/0.00	0.00/0.00
B. malayi	100/47.67	-	-	-
Cx. quinquefasciatus	100/710.19	100/3182.37	-	-
D. immitis	99.85/3.91	-	-	-
